# Variation in neurosurgical management of traumatic brain injury: a survey in 68 centers participating in the CENTER-TBI study

**DOI:** 10.1007/s00701-018-3761-z

**Published:** 2018-12-19

**Authors:** Thomas A. van Essen, Hugo F. den Boogert, Maryse C. Cnossen, Godard C. W. de Ruiter, Iain Haitsma, Suzanne Polinder, Ewout W. Steyerberg, David Menon, Andrew I. R. Maas, Hester F. Lingsma, Wilco C. Peul, on behalf of the CENTER-TBI Investigators and Participants, Ackerlund Cecilia, Adams Hadie, Agnoletti Vanni, Allanson Judith, Amrein Krisztina, Andaluz Norberto, Andelic Nada, Andreassen Lasse, Antun Azasevac, Anke Audny, Antoni Anna, Ardon Hilko, Audibert Gérard, Auslands Kaspars, Azouvi Philippe, Azzolini Maria Luisa, Baciu Camelia, Badenes Rafael, Bartels Ronald, Barzó Pál, Bauerfeind Ursula, Beauvais Romuald, Beer Ronny, Belda Francisco Javier, Bellander Bo-Michael, Belli Antonio, Bellier Rémy, Benali Habib, Benard Thierry, Berardino Maurizio, Beretta Luigi, Beynon Christopher, Bilotta Federico, Binder Harald, Biqiri Erta, Blaabjerg Morten, Boogert den Hugo, Bouzat Pierre, Bragge Peter, Brazinova Alexandra, Brinck Vibeke, Brooker Joanne, Brorsson Camilla, Buki Andras, Bullinger Monika, Calappi Emiliana, Calvi Maria Rosa, Cameron Peter, Carbayo Lozano Guillermo, Carbonara Marco, Carise Elsa, K. Carpenter, M. Castaño-León Ana, Causin Francesco, Chevallard Giorgio, Chieregato Arturo, Citerio Giuseppe, Cnossen Maryse, Coburn Mark, Coles Jonathan, Coles-Kemp Lizzie, Collett Johnny, D. Cooper Jamie, Correia Marta, Covic Amra, Curry Nicola, Czeiter Endre, Czosnyka Marek, Dahyot-Fizelier Claire, Damas François, Damas Pierre, Dawes Helen, De Keyser Véronique, Della Corte Francesco, Depreitere Bart, C. W. de Ruiter Godard, Dilvesi Dula, Ding Shenghao, Dippel Diederik, Dixit Abhishek, Donoghue Emma, Dreier Jens, Dulière Guy-Loup, Eapen George, Engemann Heiko, Ercole Ari, Esser Patrick, Ezer Erzsébet, Fabricius Martin, L. Feigin Valery, Feng Junfeng, Foks Kelly, Fossi Francesca, Francony Gilles, Freo Ulderico, Frisvold Shirin, Furmanov Alex, Gagliardo Pablo, Galanaud Damien, Gantner Dashiell, Gao Guoyi, Geleijns Karin, George Pradeep, Ghuysen Alexandre, Giga Lelde, Giraud Benoit, Glocker Ben, Golubovic Jagos, A. Gomez Pedro, Grossi Francesca, L. Gruen Russell, Gupta Deepak, A. Haagsma Juanita, Haitsma Iain, A. Hartings Jed, Helbok Raimund, Helseth Eirik, Hertle Daniel, Hoedemaekers Astrid, Hoefer Stefan, Horton Lindsay, Huijben Jilske, J. Hutchinson Peter, Håberg Asta Kristine, Jacobs Bram, Jankowski Stefan, Jarrett Mike, Jelaca Bojan, Jiang Ji-yao, Jones Kelly, Kamnitsas Konstantinos, Karan Mladen, Katila Ari, Kaukonen Maija, Kerforne Thomas, Kivisaari Riku, G. Kolias Angelos, Kolumbán Bálint, Kompanje Erwin, Kolundzija Ksenija, Kondziella Daniel, Koskinen Lars-Owe, Kovács Noémi, Lagares Alfonso, Lanyon Linda, Laureys Steven, Lecky Fiona, Ledig Christian, Lefering Rolf, Legrand Valerie, Lei Jin, Levi Leon, Lightfoot Roger, Lingsma Hester, Loeckx Dirk, Lozano Angels, I. R. Maas Andrew, MacDonald Stephen, Maegele Marc, Majdan Marek, Major Sebastian, Manara Alex, Manley Geoffrey, Martin Didier, Martin Leon Francisco, Martino Costanza, Maruenda Armando, Maréchal Hugues, Masala Alessandro, Mattern Julia, McFadyen Charles, McMahon Catherine, Melegh Béla, Menon David, Menovsky Tomas, Morganti-Kossmann Cristina, Mulazzi Davide, Muraleedharan Visakh, Murray Lynnette, Mühlan Holger, Nair Nandesh, Negru Ancuta, Nelson David, Newcombe Virginia, Nieboer Daan, Noirhomme Quentin, Nyirádi József, Oddo Mauro, Oldenbeuving Annemarie, Oresic Matej, Ortolano Fabrizio, Palotie Aarno, M. Parizel Paul, Patruno Adriana, Payen Jean-François, Perera Natascha, Perlbarg Vincent, Persona Paolo, Peul Wilco, Piippo-Karjalainen Anna, Pili Floury Sébastien, Pirinen Matti, Ples Horia, Poca Maria Antonia, Polinder Suzanne, Pomposo Inigo, Posti Jussi, Puybasset Louis, Radoi Andreea, Ragauskas Arminas, Raj Rahul, Rambadagalla Malinka, Real Ruben, Rehorčíková Veronika, Rhodes Jonathan, Ripatti Samuli, Rocka Saulius, Roe Cecilie, Roise Olav, Roks Gerwin, Rosand Jonathan, Rosenfeld Jeffrey, Rosenlund Christina, Rosenthal Guy, Rossaint Rolf, Rossi Sandra, Rueckert Daniel, Rusnák Martin, Sacchi Marco, Sahakian Barbara, Sahuquillo Juan, Sakowitz Oliver, Sala Francesca, Sanchez-Porras Renan, Sandor Janos, Santos Edgar, Sasu Luminita, Savo Davide, Schäffer Nadine, Schipper Inger, Schlößer Barbara, Schmidt Silke, Schoechl Herbert, Schoonman Guus, Schou Rico Frederik, Schwendenwein Elisabeth, Schöll Michael, Sir Özcan, Skandsen Toril, Smakman Lidwien, Smeets Dirk, Smielewski Peter, Sorinola Abayomi, Stamatakis Emmanuel, Stanworth Simon, Steinbüchel Nicole, Stevanovic Ana, Stevens Robert, Stewart William, W. Steyerberg Ewout, Stocchetti Nino, Sundström Nina, Synnot Anneliese, Taccone Fabio Silvio, Takala Riikka, Tamás Viktória, Tanskanen Päivi, Taylor Mark Steven, Te Ao Braden, Tenovuo Olli, Telgmann Ralph, Teodorani Guido, Theadom Alice, Thomas Matt, Tibboel Dick, Tolias Christos, Tshibanda Jean-Flory Luaba, Trapani Tony, Tudora Cristina Maria, Vajkoczy Peter, Vallance Shirley, Valeinis Egils, Van der Steen Gregory, Jagt van der Mathieu, Naalt van der Joukje, T. J. M. van Dijck Jeroen, A. van Essen Thomas, Van Hecke Wim, Heugten van Caroline, Van Praag Dominique, Vande Vyvere Thijs, Van Waesberghe Julia, Vanhaudenhuyse Audrey, Vargiolu Alessia, Vega Emmanuel, Velt Kimberley, Verheyden Jan, M. Vespa Paul, Vik Anne, Vilcinis Rimantas, Vizzino Giacinta, Vleggeert-Lankamp Carmen, Volovici Victor, Voormolen Daphne, Vulekovic Peter, Vámos Zoltán, Wade Derick, K. W. Wang Kevin, Wang Lei, Wessels Lars, Wildschut Eno, Williams Guy, Wilson Lindsay, K. L. Winkler Maren, Wolf Stefan, Ylén Peter, Younsi Alexander, Zaaroor Menashe, Zhihui Yang, Ziverte Agate, Zumbo Fabrizio

**Affiliations:** 1 Department of Neurosurgery, Leiden University Medical Center, University Neurosurgical Center Holland (UNCH), Leiden, The Netherlands; 2 Department of Neurosurgery, Haaglanden Medical Center, University Neurosurgical Center Holland (UNCH), The Hague, The Netherlands; 3 Department of Neurosurgery, Radboud University Medical Center, Nijmegen, The Netherlands; 4 Center for Medical Decision Sciences, Department of Public Health, Erasmus Medical Center, Rotterdam, The Netherlands; 5 Department of Neurosurgery, Erasmus Medical Center, Rotterdam, The Netherlands; 6 Department of Biomedical Data Sciences, Leiden University Medical Center, Leiden, The Netherlands; 7 Division of Anaesthesia, Addenbrooke’s Hospital, University of Cambridge, Cambridge, UK; 8 Department of Neurosurgery, Antwerp University Hospital and University of Antwerp, Edegem, Belgium

**Keywords:** Traumatic brain injury, Neurosurgery, Practice variation, Acute subdural hematoma

## Abstract

**Background:**

Neurosurgical management of traumatic brain injury (TBI) is challenging, with only low-quality evidence. We aimed to explore differences in neurosurgical strategies for TBI across Europe.

**Methods:**

A survey was sent to 68 centers participating in the Collaborative European Neurotrauma Effectiveness Research in Traumatic Brain Injury (CENTER-TBI) study. The questionnaire contained 21 questions, including the decision when to operate (or not) on traumatic acute subdural hematoma (ASDH) and intracerebral hematoma (ICH), and when to perform a decompressive craniectomy (DC) in raised intracranial pressure (ICP).

**Results:**

The survey was completed by 68 centers (100%). On average, 10 neurosurgeons work in each trauma center. In all centers, a neurosurgeon was available within 30 min. Forty percent of responders reported a thickness or volume threshold for evacuation of an ASDH. Most responders (78%) decide on a primary DC in evacuating an ASDH during the operation, when swelling is present. For ICH, 3% would perform an evacuation directly to prevent secondary deterioration and 66% only in case of clinical deterioration. Most respondents (91%) reported to consider a DC for refractory high ICP. The reported cut-off ICP for DC in refractory high ICP, however, differed: 60% uses 25 mmHg, 18% 30 mmHg, and 17% 20 mmHg. Treatment strategies varied substantially between regions, specifically for the threshold for ASDH surgery and DC for refractory raised ICP. Also within center variation was present: 31% reported variation within the hospital for inserting an ICP monitor and 43% for evacuating mass lesions.

**Conclusion:**

Despite a homogeneous organization, considerable practice variation exists of neurosurgical strategies for TBI in Europe. These results provide an incentive for comparative effectiveness research to determine elements of effective neurosurgical care.

**Electronic supplementary material:**

The online version of this article (10.1007/s00701-018-3761-z) contains supplementary material, which is available to authorized users.

Neurosurgical decision-making in patients with traumatic brain injury (TBI) is often challenging for several reasons. First, no two TBI patients are identical—clinical and radiological findings may differ greatly [26]. Second, there is no high-quality evidence to support the range of possible neurosurgical procedures in TBI. Indications for surgical management are summarized in the Brain Trauma Foundation guidelines, [5] but are merely based on retrospective studies of small groups of selected patients. These guidelines provide general advice on surgical indications for evacuation of acute epidural (EDH), acute subdural (ASDH), and contusions/intracerebral hematomas (ICH) based on the size of the hematoma and midline shift. The guidance for decompressive surgery is even less clear. It is mostly performed to decrease raised intracranial pressure (ICP), either as a primary procedure in an acute setting, or as a secondary procedure to deal with diffuse edema or peri-contusional swelling. The guidelines state that this latter use of secondary decompression can reduce ICP, but does not necessarily improve outcome [6]. More fundamentally, the rationale for ICP monitoring has been challenged by the BEST TRIP randomized controlled trial (RCT), which found no benefit of a management protocol based on intracranial pressure monitoring, compared to one based on serial imaging and clinical examination. These results have generated doubts regarding ICP monitoring [1, 7, 15, 20, 28]. Overall, there is no clear consensus on the indications, extent, and timing of surgery [32].

This limited high-quality evidence for surgical management in TBI arises from a lack of RCTs, which may be difficult to conduct due to pragmatic, ethical, and methodological barriers [3]; however, observational studies to determine effectiveness are more prone for bias [2]. A promising alternative approach could be comparative effectiveness research (CER) [24, 33]. In this design, the heterogeneity and variability, that trouble RCTs in TBI, are accepted and exploited to study effectiveness of treatments as they occur in real-life practice. The current Collaborative European Neurotrauma Effectiveness Research in Traumatic Brain Injury (CENTER-TBI) study aims to use CER methodology to study treatment effectiveness of several neurosurgical interventions [25].

The aim of this study was to explore differences in neurosurgical strategies for TBI across Europe to provide a context for CENTER-TBI, an up-to-date insight into European neurosurgical management of TBI, and to identify naturally occurring variation between trauma centers in order to identify substrates for neurosurgical research questions that might be answered using CER in the study.

## Materials and methods

**Fig. 1:**
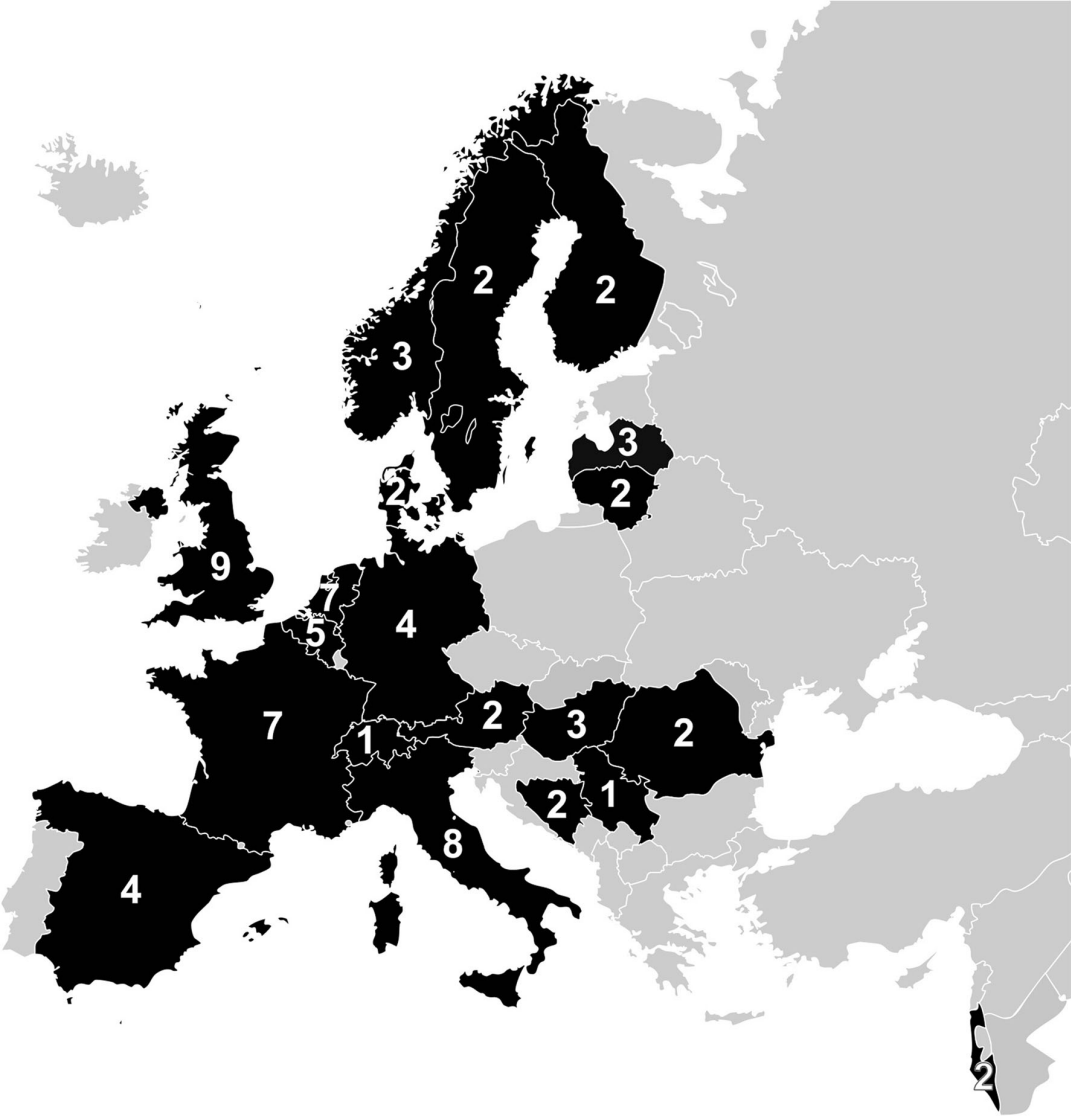
Centers and countries included in the Collaborative European NeuroTrauma Effectiveness Research in Traumatic Brain Injury (CENTER-TBI) study. Reprinted and updated from Cnossen et al. (2016) with permission from Dr. Cnossen and Maas et al. (2015). Collaborative European NeuroTrauma Effectiveness Research in Traumatic Brain Injury: a prospective longitudinal observational study. Neurosurgery, 76:67–80, under a CC BY license, with permission from professor A.I. Maas

This study was conducted within the setting of the international observational study CENTER-TBI [25]. Between 2014 and 2015, all centers participating in the international multicenter observational study CENTER-TBI (www.CENTER-TBI.eu) were asked to complete a questionnaire on neurosurgical management of TBI (Supplementary file 1) [9]. The questionnaire was sent to 71 centers (Fig. 1), of which five centers dropped out and two joined in, resulting in 68 eligible centers from Austria (*n* = 2), Belgium (*n* = 4), Bosnia Herzegovina (*n* = 2), Denmark (n = 2), Finland (n = 2), France (*n* = 7), Germany (n = 4), Hungary (*n* = 3), Israel (n = 2), Italy (*n* = 10), Latvia (n = 3), Lithuania (n = 2), Norway (n = 3), Romania (n = 1), Serbia (n = 1), Spain (n = 4), Sweden (n = 2), Switzerland (n = 1), The Netherlands (*n* = 6), and The United Kingdom (*n* = 7).

### Questionnaire development and administration

We developed a set of questionnaires based on available literature and experts to measure the structure and processes of TBI care in individual centers. Details regarding this process and the questionnaires used are described in a separate paper [9]. Pilot testing was undertaken in 16 of the participating centers and feedback was incorporated into the final design.

One of the questionnaires was on neurosurgical standard practice. This survey contained 21 questions which could broadly be divided into 3 categories: (1) center characteristics and internal structure; (2) general (neuro) surgical trauma care and processes; and (3) site specific neurosurgical management for treating ASDH, EDH, ICH, the use of DC, and policy with regard to orthopedic injuries in the context of patients who had suffered a TBI.

Questions either sought quantitative estimates of key metrics (e.g., annual surgical volume, staff size, ASDH thickness, or ICP thresholds for surgery) or attempted to elicit the “general policy” of the center. To capture the latter, these questions were formulated in two ways: respondents were asked to estimate what the management strategy is in more than three quarters of patients in their center in a given context; or respondents were asked to indicate how often they used a particular surgical technique or how often specific factors influence their decision-making (never = 0–10%, rarely = 10–30%, sometimes = 30–70%, frequently = 70–90%, and always 90–100%). The options “frequently” and “always” were interpreted as “general policy”, in line with a previous report [17] and similar to previous publications on other questionnaires [8, 9].

The reliability of the surveys was tested by calculation of concordance in a previous publication [9]. Overall, the median concordance rates between duplicate questions were 0.81 (range 0.44–0.97) and specifically for the “Neurosurgery” survey 0.78 (range 0.68–0.86).

### Analyses

The median and interquartile range (IQR) were calculated for continuous variables, and frequencies were reported along with percentages for categorical variables. Countries were divided into seven geographic regions: Northern Europe (Norway 3, Sweden 2, Finland 2 and Denmark 2 centers), Western Europe (Austria 2, Belgium 4, France 7, Germany 4, Switzerland 1 and The Netherlands 6 centers), The United Kingdom (7 centers), Southern Europe (Italy 10 and Spain 4 centers), Eastern Europe (Hungary 3, Romania 1, Serbia 1 and Bosnia Herzegovina 2 centers), Baltic States (Latvia 3 and Lithuania 2 centers), and Israel (2 centers).

For the following neurosurgical treatment strategies, we quantified regional differences: an absolute cutoff of hematoma thickness as an indication for surgery for ASDH, DC in the primary evacuation of an ASDH, early/pre-emptive surgical evacuation for ICH, and DC as a general policy in case of refractory raised ICP.

To assess the association of region with one of these treatment choices, a logistic regression was performed with treatment choice (general policy or “yes/no”) as a dependent variable and the region (categorical) as independent variable. Nagelkerke R2 indicated the variance explained by geographic region. Analyses were done in IBM SPSS Statistics version 20 (IBM, Chicago, IL, USA).

## Results

### Center characteristics

All 68 eligible centers completed the questionnaire on neurosurgery (response rate 100%). Questionnaires were mainly completed by neurosurgeons (*n* = 53, 78%), followed by local CENTER-TBI investigators (mainly research physicians or nurses: 19%). On average, 10 neurosurgeons (IQR 8–13) and four trauma surgeons (IQR 0–12) worked in each center. All centers reported that neurosurgical coverage was available 24 h a day/7 days a week, either by way of in-house availability of a qualified neurosurgeon (47%), or the availability of such an individual in less than 30 min (53%) (Table 1).

**Table 1:** Characteristics of centers participating in neurosurgery survey

Characteristic	*N* completed	No. (%) or median (IQR)
Profession of respondent	68	
Neurologist		3 (4)
Neurosurgeon		53 (78)
Trauma surgeon		3 (4)
ED physician		1 (2)
Intensivist		1 (1)^a^
Administrative staff member		11 (16)^a^
CENTER-TBI local investigator		13 (19)^a^
Volume of surgeries in 2013^c^		
ASDH	59	25 (15–49)
ICH/contusion	58	10 (5–21)
EDH	59	10 (5–19)
DC		
Hemicraniectomy	57	10 (5–16)
Bifrontal	57	0 (0–2)
Removal bone flap	55	1 (0–3)
Ventriculostomy	57	7 (2–21)
Cranioplasty	56	10 (6–14)
Depressed skull fracture	57	5 (2–12)
Staffing (FTE)		
Neurosurgeons	66	10 (8–13)
Residents in training	65	5 (3–8)
Residents not in training	61	0 (0–3)
Trauma surgeons	64	4 (0–12)
Organization of care		
Neurosurgical decision making in ICU	68	
Neurosurgeon		65 (96)
Trauma surgeon		1 (3)
Neurologist		0
Neurointensivist or general intensivist		1 (2)
24/7 neurosurgical coverage^b^	68	
Qualified neurosurgeon in-house		32 (47)
Resident neurosurgery in-house		30 (44)
Neurosurgeon within 30 min		36 (53)
Neurosurgical resident within 30 min		11 (16)
Neurosurgeon more than 30 min		0 (0)

*ASDH* acute subdural hematoma, *EDH* epidural hematoma, *ICH* intracerebral hematoma, *DC* decompressive craniectomy, *FTE* full time equivalent, *ICU* intensive care unit

^a^Numbers do not add up because the local investigators also depicted their profession and one responder declared to be an intensivist as well as an administrative staff member

^b^Multiple options possible

^c^Head trauma–related surgeries

### General (neuro) surgical care and processes

**Fig. 2:**
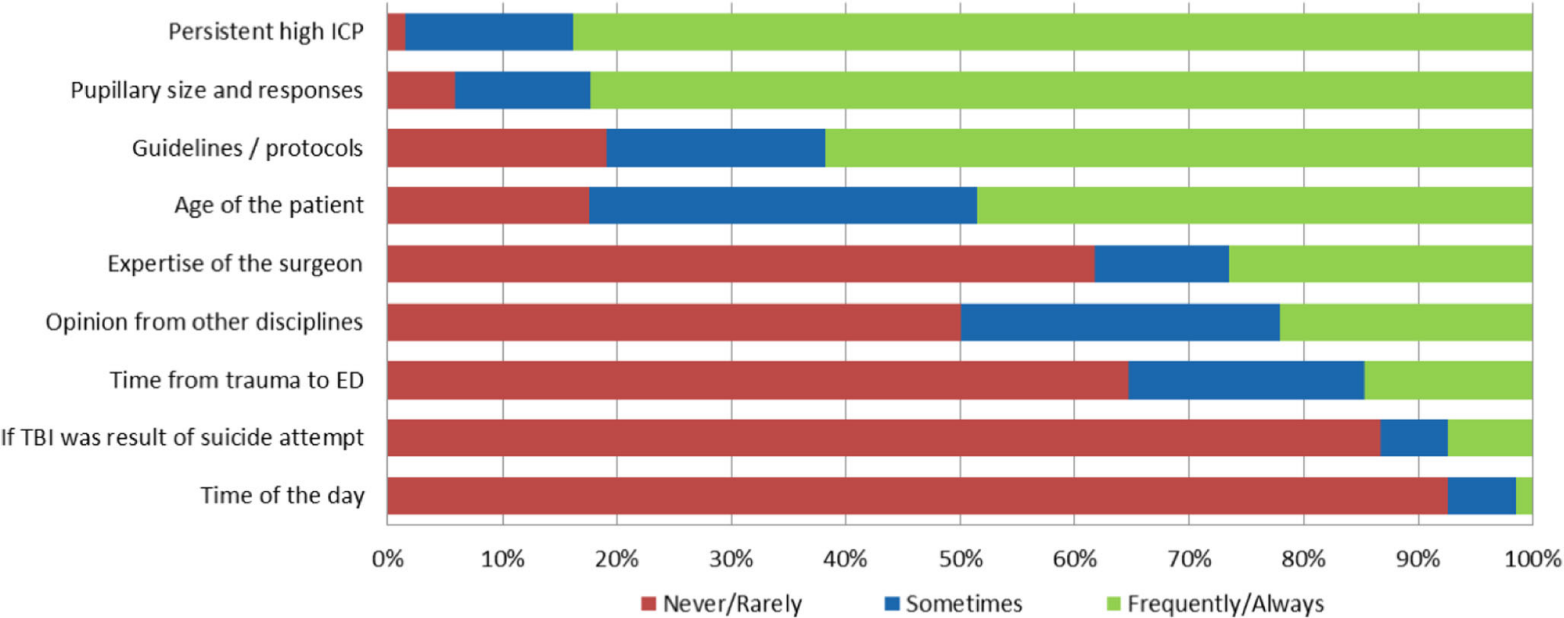
Factors of influence on neurosurgical decision-making. Shown are the percentages of centers that would be never/rarely, sometimes or frequently/always influenced by the described factors in the decision to perform neurosurgical procedures. Question was completed by all 68 centers. ICP: intracranial pressure; ED: Emergency Department ^B^ Other factors were not predetermined but were specified by responders

Treatment decisions regarding cranial surgical interventions in TBI patients within the critical care ER and ICU period are in most centers determined by the neurosurgeon (*n* = 65, 96%), followed by the orthopedic surgeons and neuro-intensivist in respectively 3% (*n* = 2) and 1% (*n* = 1). Urgent neurosurgical interventions (ICP monitor device insertion not included) for life-threatening traumatic intracranial lesions, are made by the neurosurgeon in 98.5% and trauma surgeons in 1.5% of the centers. Raised ICP will almost always be incorporated in decision-making, the time of day almost never (Fig. 2).

With regard to extremity fractures, the general policy in 59 (87%) centers was so-called damage control with priority for TBI and delayed definitive treatment of the limb fractures (Table 2). This policy is protocolized in 21 centers (22%).

**Table 2:** Neurosurgical treatment policy of traumatic brain injury

Characteristic	*N* completed	No. (%) or mean (sd)
Structural estimation of mass lesions on CT^a^	68	
Visual intuition (e.g., no actual measurement)		27 (40)
Width, diameter and/or amount of MLS of the mass lesion		58 (85)
Volume measurements with imaging software		11 (16)
Volume measurements with direct calculation		17 (25)
Other		1 (2)
ASDH operation determinants		
Age considered important in surgery decision^d^	68	26 (42)
Size (volume or thickness) threshold for surgery	68	27 (40)
Minimum volume or thickness:	28^b^	
15 mm		2 (3)
10 mm		16 (24)
10 mm and/or > 5 mm MLS		2 (3)
5 mm		3 (4)
ASDH thickness > width of cranium		3 (4)
Midline shift > thickness ASDH		2 (3)
DC indications	68	
Routine		4 (6)
Intra-operative brain swelling		59 (86)
Sometimes as a second procedure in case of uncontrollable ICP		5 (7)
Never		0 (0)
ICH/contusion operation determinants		
General policy	68	
Pre-emptive (to prevent deterioration)		2 (3)
Delayed (after deterioration)		45 (66)
Variable (depends on surgeon)		18 (27)
Other		3 (4)
DC indications	68	
Routine		1 (2)
Intra-operative brain swelling		55 (81)
Sometimes as a delayed procedure in case of uncontrollable ICP		10 (15)
Never		2 (3)
Raised ICP determinants		
DC employed > 70% of refractory high ICP cases	68	32 (46)
Mostly early DC (within 6–12 h of refractory ICP)	64	32 (47)
Mostly late DC (as last resort to control ICP)	64	32 (47)
ICP threshold for DC	68	65 (96)
Raised ICP threshold for DC (mmHg):	64^c^	
30		12 (18)
25		39 (60)
20		11 (17)
15		1 (2)
Not standardized		1 (2)
DC indications considered^a^		
Pre-emptive in raised ICP (not last resort)		7 (10)
Refractory raised ICP (last resort)	68	64 (91)
CT evidence of raised ICP		9 (13)
Intra-operative brain swelling		45 (66)
Routine with every ASDH or ICH evacuation		2 (3)
Policy towards extremity limb fractures^e^		
Damage control		59 (87)
Definitive care	68	9 (13)

*MLS* midline shift, *BTF* Brain Trauma Foundation, *ICP* intracranial pressure, *hrs* hours

^a^Multiple options possible

^b^One responder did not report a threshold for surgery while answering a specific threshold (10 mm)

^c^One responder reported to employ a threshold for DC in raised ICP while not giving their specific threshold

^d^The question was whether the responder considers if the decision on surgery in acute SDH is influenced by age (based on a general consensus in their respective center)

^e^Damage control is focused on the TBI. All extremity fractures are stabilized, but definitive treatment delayed. Definitive care: the extremity fractures are operated as soon as possible

Of all centers, 58 (85%) estimated the space-occupying effect of traumatic lesions on the surrounding tissue by calculation of the thickness of the hematoma and midline shift on CT. A quarter of centers used actual volume measurement to make surgical decisions (Table 2).

### Neurosurgical management of ASDH, EDH, ICH, and the use of decompressive craniectomy

ASDH provided the highest volume of neurosurgical TBI cases, on average 25 cases per year. When performing a DC (for any indication), hemicraniectomy was the preferential technique, and bifrontal craniectomy was rarely performed (Table 1). Less than half of the centers (*n* = 27, 40%) reported an absolute threshold for evacuating an ASDH. Four out of 10 centers generally incorporate age in their decision for evacuating an ASDH (Table 2 and Fig. 2).

ICH were seldom operated upon pre-emptively, but 67% of centers reported undertaking delayed surgery in the event of deterioration. Almost a third of centers reported within-center variations between individual neurosurgeons in decisions regarding surgical evacuation of contusions or traumatic ICH.

**Fig. 3:**
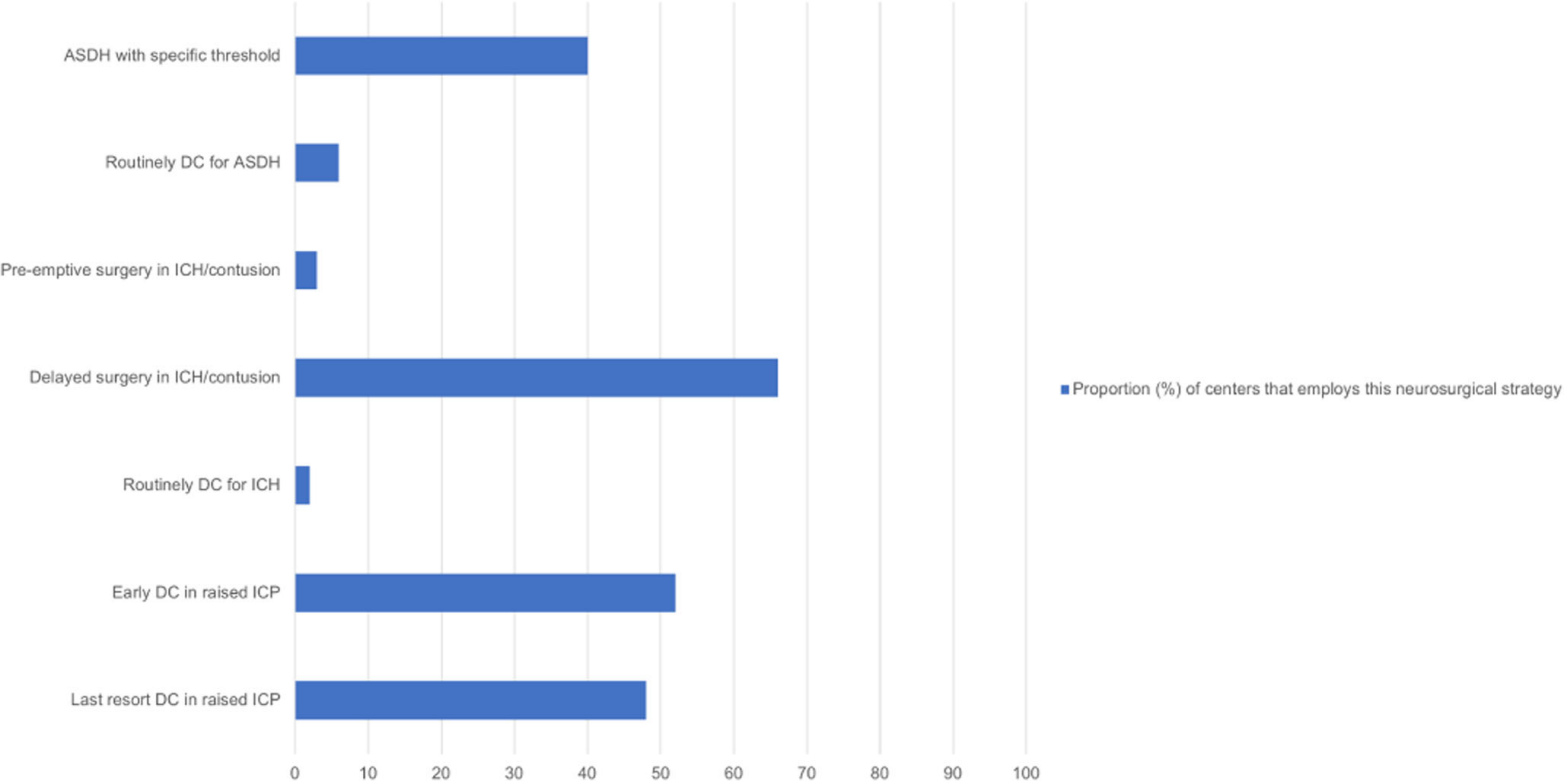
Treatment indications for neurosurgical interventions. Shown are the proportions of centers that generally have these specific preferences with regard to operating or not in ASDH, ICH, and raised intracranial pressure, respectively. ASDH: acute subdural hematoma; DC: decompressive craniectomy; ICH: intracerebral hematoma; ICP: intracranial pressure

Only a very low proportion of centers would routinely perform a DC at the time of evacuation of either ASDH or ICH (respectively 6% and 1.5% of the centers). For refractory raised ICP, most centers (*n* = 64, 91%) would consider a decompressive craniectomy, while 32 (47%) see this as a general policy in their center (Fig. 3, Table 2 and figure in supplementary file 2). Ninety-six percent (*n* = 65) reported to have a specific threshold for DC in refractory raised ICP. This was most commonly specified as 25 mmHg (*n* = 39, 58%), followed by 30 mmHg (*n* = 12, 18%) and 20 mmHg (*n* = 11, 17%).

### Guidelines and practice variation

**Fig. 4:**
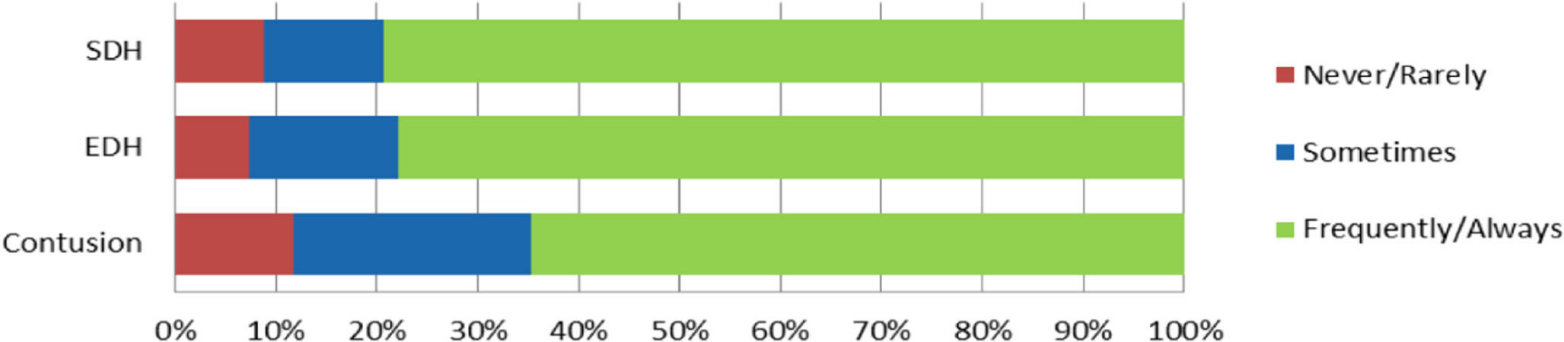
BTF guideline adherence. Shown are the percentages of centers that reported to never/rarely, sometimes or frequently/always follow the Brain Trauma Foundation guidelines for the management of SDH, EDH, or contusions. Question was completed by 68 of the 68 centers. TBI: traumatic brain injury; SDH: subdural hematoma; EDH: epidural hematoma

Overall, the reported adherence to the BTF guidelines was high (Fig. 4). The use of surgical interventions and specific indications for these interventions varied substantially within and between regions (Table 3). Surgical evacuation of ICH was only performed in the Baltic States and Southern Europe and geographic region explained 35% of the variance in use of the intervention. Having a specific threshold for ASDH surgery and employing a DC for refractory-raised ICP showed the largest within-region and also between-region variation. Lastly, when directly asked whether variation in specific management strategies exist, respectively 31% and 43% indicated to have a structural variation within their center staff with regard to ICP sensor insertion and mass lesion evacuation (Table 4).

**Table 3:** Within- and between-region variation in surgical management

Decision	Northern Europe	Western Europe	United Kingdom	Southern Europe	Eastern Europe	Baltic States	Israel	Nagelkerke R^2^ value
*ASDH*								
- Size threshold for evacuation	56	29	0	29	71	80	100	0.34
- Routine or intraoperative DC *ICH/contusion*	89	92	100	100	86	80	100	0.17
- Pre-emptive surgery *Refractory raised ICP*	0	0	0	7	0	20	0	0.35
- DC	44	37	29	57	43	80	100	0.15

*ASDH* acute subdural hematoma, *ICH* intracerebral hematoma, *DC* decompressive craniectomy, *ICP* intracranial pressure

Table presents the proportion (%) of respondent within each region that indicated that they used the described strategy as their general policy for patients with respectively ASDH, ICH, or refractory raised ICP. The Nagelkerke R^2^ value represents the variation in treatment that can be explained by the region

**Table 4:** Neurosurgical decision making

Characteristic	*N* completed	No (%)
Structural variation^a^ ICP monitor insertion	68	
No		47 (69)
Yes		21 (31)
Structural variation^a^ mass lesion evacuation	65	
No		29 (43)
Yes		29 (43)
Depending on lesion type		7 (10)

*ED* emergency department, *GCS* Glasgow Coma Scale

^a^Structural variation refers to a situation in which one or more of the clinicians are generally more likely to perform the (diagnostic) intervention than others

## Discussion

The aim of this study was to explore differences in neurosurgical strategies for TBI across Europe. We found substantial variability in practice and thereby provide useful indications regarding potential substrates for CER in CENTER-TBI. The structures and processes of neurosurgical care are generally homogeneous across centers with a comparable number of neurosurgeons, similar organization of neurosurgical coverage and uniform organization of responsibility for most surgical decisions on the ER and ICU. The indications for surgery, however, differ substantially with high within-region and between-region practice variations.

### Contemporary neurosurgical care

There are no recent comparable studies providing an overview of neurosurgical management on this scale. Two recent national surveys, in The United Kingdom and the Republic of Ireland and The Netherlands, have shown a comparable variability among neurosurgeons regarding the decision to evacuate an ASDH or to perform a primary DC [21, 34].

When comparing our results to existing—much older—surveys, evacuation of a traumatic ICH seems to be less often considered than in the past [11, 30]. Our results are concordant with older surveys in reporting variable use of DC for refractory raised ICP, despite the DECRA trial (the RECUEicp was not published yet) [12, 19]. Interestingly, although the mostly applied cutoff for DC in refractory is reported to be 25 mmHg (60%), a lower value, 20 mmHg, and a higher value, 30 mmHg, are both reported to be used in almost 20% of centers.

More broadly, our results replicate past data that suggest poor guideline adherence and practice variability. Rayan et al. showed that in only 17% of a random sample of (brain) trauma patients care was delivered according to the BTF guidelines [31]. Of note, in the current study, surveys were sent to the centers between 2014 and 2015, so the more recent, updated BTF guidelines were not published yet, although the update was for medical management mainly (except DC in refractory IC) [6].

Comparable questionnaires on other aspects of TBI care have recently been published for ER and ICU management that, without exception, show practice variation [8, 9, 14, 18]. Practice variation has also been reported for other life-threatening or emergency disorders including ruptured abdominal aneurysm [4] and the spontaneous intracerebral hemorrhage [16].

### Strengths and limitations

A strength of the current study is the methodology that we used to investigate practice variation. First, detailed questions were posed to shed light on specific clinical decisions with regard to neurosurgical interventions. Subsequently, (objective) answers on amounts (volume load, mostly from in-hospital registries) were combined with qualitative information (estimations of general policies, using two approaches). When integrated with the high response rate and low amount of missing data in 68 centers, this overview provides a complete picture of reported neurosurgical care across Europe.

This study also had weaknesses. First, responses to the questionnaire may have been biased by the abstract nature of the questions posed, which neglected to provide a more concrete clinical context for judgments about reported practice. Although the respondents were experienced neurosurgeons with a scientific background, the difficulty of weighing individual patient characteristics with potentially fatal consequences can never be fully captured by a theoretical survey. In particular, the rational decision-making can obviously be completely different due to the cognitive biases of neurosurgeons in the acute critical care period.

Second, there might be a concern as to how well the individual neurosurgeon respondent can represent the general center neurosurgical policy. Although we urged the respondent to report the general consensus on treatment at their center rather than individual management preferences (see Supplementary file 1), neurosurgical strategies may still be variable within centers between neurosurgeons; however, we did capture a qualitative assessment of this intra-center variability (Table 4). Third, we did not fully account for inherent regional variations such as evidence knowledge, caseload, and case-mix due to referral patterns or admission policies, as a potential explanation for differences in neurosurgery policies. Variations in evidence knowledge for some questions, such as those on guidelines, are important. Moreover, while we did asses the center’s caseload and casemix, the caseload and casemix of the (individual) respondent was not specifically asked. Fourth, the questions dealt with individual decisions in isolation, rather than the more complex real-life situation where several competing priorities need to be addressed. Fifth, the reports may have been biased (in varying extents) towards how centers would have been liked to be perceived, rather than a faithful report of actual clinical policy and practice. This issue will be addressed by a planned comparison of these Provider Profiling responses with actual treatment strategies employed in patient-level data from these centers in the CENTER-TBI Core study.

Finally, our study sample represents centers participating in TBI-research which are likely specialized neurotrauma centers with a tendency to have practice that is skewed towards up-to-date knowledge. An example is the fact that almost half of all centers stated to have a neurosurgeon in house 24 h a day. When studying all centers in Europe providing care to TBI patients, variability might be even larger.

### Implications

Our results should be interpreted in combination with the current evidence on the effectiveness of different surgical strategies. For the use of DC in refractory raised ICP due to diffuse swelling, two RCTs have provided useful guidance. The DECRA trial showed that early use of DC for modest rises in ICP was associated with worse outcomes [12]. More recently however, after the conduct of this survey, the RESCUEicp trial showed that, when used for refractory severe intracranial hypertension, DC can save lives, but results in an excess of severely disabled survivors [19]. It is clear that the intervention is not uniformly beneficial: while some functional improvements occur by 12 months, many survivors remain severely disabled. Rescue-ICP was not published yet at the conduct of this study. In our study, the majority of centers indicated that DC is often employed for both indications (pre-emptive and last resort).

With regard to focal lesions, a recent study suggested that in patients with an ASDH an aggressive approach towards evacuation is associated with better outcome compared to a conservative approach [35]. Similar trends were noted in the STITCH-trauma trial, which suggested better outcome with early surgical management of ICH [29]. In our study, a minority of centers considers an early strategy for ICH evacuation.

Lastly, DC in the primary evacuation of an ASDH seems to be associated with more favorable outcomes [22]. There is no class 1 evidence, although the research question is currently being challenged in an RCT (Rescue-ASDH; ISRCT87370545). In the current survey standard (in some cases preventive) DC in ASDH evacuation is rarely employed but mostly done in case of intraoperative swelling.

There may be several explanations for the practice variation that we observed. Although high practice variation rates can be a sign of poor implementation of evidence-based care, in this context it probably reflects the lack of strong evidence to underpin practice. In such a low evidence context, clinical decisions are not driven by careful consideration or penetration of the evidence, but by local customs and surgical training, handed down over the years from one surgeon to the other in a given center (or country). The professional cultural drivers that underpin such learned treatment preferences are resistant to change, and provide an important hurdle to the design and conduct of randomized studies for neurosurgical interventions in TBI [27].

Additionally, even where the results of RCTs are available, it is possible that many neurosurgeons do not think the RCT results applicable to their (individual) patients, or restrict their focus to short term clinical outcomes such as mortality and complication rates (instead of long-term clinical or patient reported outcomes). [13]

The results of the questionnaire point out burning clinical questions for neurosurgery in TBI. For ASDH and ICH, important questions include whether to operate or not, the timing of operative evacuation, and whether or not a primary DC should be undertaken. Future studies should address these questions. For DC, the variation should lead to studies exploring the lack of evidence penetration, in addition to studying effectiveness of DC in refractory raised ICP.

While RCTs may provide the security of randomization as a basis for examining answering these questions, RCTs have no successful history in TBI due to various reasons [24]. The CENTER-TBI Provider Profiling exercise has revealed large practice variation that can be related to variation in patient outcome [23]. Such a CER approach may be a pragmatic alternative to RCTs.

Therefore, different steps are required. Firstly, to specify, ideally a-priori, how and where treatment variation occurs. This was one of the goals of this provider profiling. Secondly, the CENTER-TBI Core Study will need to collect patient-level data from a large variety of centers, capturing the range of treatment variation and relate it to outcome. The main challenge is to disentangle the effect of specific surgical strategies in a center from other regional care variation that might affect outcome. To do so, we propose random-effect models in which the effect of “surgical strategy” on outcome is estimated with adjustment for other between-hospital differences in a random effect for hospital [10, 34, 35].

## Conclusions

This survey study explored differences in neurosurgical strategies for TBI. Current neurosurgical care differs within Europe (and Israel), while the organization of trauma centers does not. This variation in practice likely reflects the lack of high-quality evidence for these important, potentially life-saving, emergency neurosurgical interventions. In addition, local professional culture may drive practice in ways that are not dependent on the availability or penetration of evidence. The resulting entrenched practice variation does not facilitate equipoise that makes RCTs easy to deliver. CER may provide a pragmatic approach to generate evidence on optimal neurosurgical strategies for TBI patients.

### Funding/sponsors

This study was funded by the European Union Seventh Framework Program (grant 602150) for Collaborative European NeuroTrauma Effectiveness Research in Traumatic Brain Injury (CENTER-TBI) and the Hersenstichting Nederland (Dutch Brain Foundation, grant PS2014-06) for The Dutch Neurotraumatology Quality Registry (Net-QuRe). There is no industry affiliation.

### Electronic supplementary material

Supplementary material 1 Questionnaire neurosurgery. (PDF 158 kb)

Supplementary Figure 1 The use of a decompressive craniectomy. (PNG 651 kb)

High resolution image (TIF 5387 kb)

Shown are the percentages of centers that would add DC for the surgical management in the following matter: yes, routinely; yes, depending on intraoperative findings; sometimes, as second surgery in case of refractory ICP; never. Also percentages are shown of the timing of DC and if centers would never/rarely, sometimes or frequently/always use DC in case of refractory ICP. Questions were completed by 68 of the 68 centers, expect for the question on early or late use of DC (no answer from four centers).

DC: decompressive craniectomy; SDH: subdural hematoma; EDH: epidural hematoma; ICP: intracranial pressure.

## Appendix

The CENTER-TBI Investigators and Participants and their affiliations:

Ackerlund Cecilia^1^, Adams Hadie ^2^, Agnoletti Vanni ^3^, Allanson Judith ^4^, Amrein Krisztina ^5^, Andaluz Norberto ^6^, Andelic Nada ^7^, Andreassen Lasse ^8^, Antun Azasevac ^9^, Anke Audny ^10^, Antoni Anna ^11^, Ardon Hilko ^12^, Audibert Gérard ^13^, Auslands Kaspars ^14^, Azouvi Philippe ^15^, Azzolini Maria Luisa ^16^, Baciu Camelia ^17^, Badenes Rafael ^18^, Bartels Ronald ^19^, Barzó Pál ^20^, Bauerfeind Ursula ^21^, Beauvais Romuald ^22^, Beer Ronny ^23^, Belda Francisco Javier ^18^, Bellander Bo-Michael ^24^, Belli Antonio^25^, Bellier Rémy ^26^, Benali Habib ^27^, Benard Thierry ^26^, Berardino Maurizio ^28^, Beretta Luigi ^16^, Beynon Christopher ^29^, Bilotta Federico ^18^, Binder Harald ^11^, Biqiri Erta ^17^, Blaabjerg Morten ^30^, den Boogert Hugo ^19^, Bouzat Pierre ^31^, Bragge Peter ^32^, Brazinova Alexandra ^33^, Brinck Vibeke ^34^, Brooker Joanne ^35^, Brorsson Camilla ^36^, Buki Andras ^37^, Bullinger Monika ^38^, Calappi Emiliana ^39^, Calvi Maria Rosa ^16^, Cameron Peter ^40^, Carbayo Lozano Guillermo ^41^, Carbonara Marco^39^, Carise Elsa ^26^, Carpenter K. ^42^, Castaño-León Ana M. ^43^, Causin Francesco ^44^, Chevallard Giorgio ^17^, Chieregato Arturo ^17^, Citerio Giuseppe ^45, 46^, Cnossen Maryse ^47^, Coburn Mark ^48^, Coles Jonathan ^49^, Coles-Kemp Lizzie ^50^, Collett Johnny ^50^, Cooper Jamie D. ^51^, Correia Marta ^52^, Covic Amra ^53^, Curry Nicola ^54^, Czeiter Endre ^55^, Czosnyka Marek ^56^, Dahyot-Fizelier Claire ^26^, Damas François ^57^, Damas Pierre ^58^, Dawes Helen ^59^, De Keyser Véronique ^60^, Della Corte Francesco ^61^, Depreitere Bart ^62^, de Ruiter Godard C.W. ^63^, Dilvesi Dula ^9^, Ding Shenghao ^64^, Dippel Diederik ^65^, Dixit Abhishek^66^, Donoghue Emma ^40^, Dreier Jens ^67^, Dulière Guy-Loup ^57^, Eapen George ^68^, Engemann Heiko ^53^, Ercole Ari ^66^, Esser Patrick ^59^, Ezer Erzsébet ^69^, Fabricius Martin ^70^, Feigin Valery L. ^71^, Feng Junfeng ^64^, Foks Kelly ^65^, Fossi Francesca ^17^, Francony Gilles ^31^, Freo Ulderico ^72^, Frisvold Shirin ^73^, Furmanov Alex ^74^, Gagliardo Pablo ^75^, Galanaud Damien ^27^, Gantner Dashiell ^40^, Gao Guoyi ^76^, Geleijns Karin ^42^, George Pradeep ^1^, Ghuysen Alexandre ^77^, Giga Lelde ^78^, Giraud Benoit ^26^, Glocker Ben ^79^, Golubovic Jagos ^9^, Gomez Pedro A. ^43^, Grossi Francesca ^61^, Gruen Russell L. ^80^, Gupta Deepak ^81^, Haagsma Juanita A. ^47^, Haitsma Iain ^82^, Hartings Jed A. ^83^, Helbok Raimund ^23^, Helseth Eirik ^84^, Hertle Daniel ^30^, Hoedemaekers Astrid ^85^, Hoefer Stefan ^53^, Horton Lindsay ^86^, Huijben Jilske ^47^, Hutchinson Peter J. ^2^, Håberg Asta Kristine ^87^, Jacobs Bram ^88^, Jankowski Stefan ^68^, Jarrett Mike ^34^, Jelaca Bojan^9^, Jiang Ji-yao ^76^, Jones Kelly ^89^, Kamnitsas Konstantinos ^79^, Karan Mladen ^6^, Katila Ari ^90^, Kaukonen Maija ^91^, Kerforne Thomas ^26^, Kivisaari Riku ^91^, Kolias Angelos G. ^2^, Kolumbán Bálint ^92^, Kompanje Erwin ^93^, Kolundzija Ksenija ^94^, Kondziella Daniel ^70^, Koskinen Lars-Owe ^36^, Kovács Noémi ^92^, Lagares Alfonso ^43^, Lanyon Linda ^1^, Laureys Steven ^95^, Lecky Fiona ^96^, Ledig Christian ^79^, Lefering Rolf ^97^, Legrand Valerie ^98^, Lei Jin ^64^, Levi Leon ^99^, Lightfoot Roger ^100^, Lingsma Hester ^47^, Loeckx Dirk ^101^, Lozano Angels ^18^, Maas Andrew I.R. ^60^, MacDonald Stephen ^102^, Maegele Marc ^103^, Majdan Marek ^33^, Major Sebastian ^104^, Manara Alex ^105^, Manley Geoffrey ^106^, Martin Didier ^107^, Martin Leon Francisco ^101^, Martino Costanza ^3^, Maruenda Armando ^18^, Maréchal Hugues ^57^, Masala Alessandro ^3^, Mattern Julia ^29^, McFadyen Charles^66^, McMahon Catherine ^108^, Melegh Béla ^109^, Menon David ^66^, Menovsky Tomas ^60^, Morganti-Kossmann Cristina ^110^, Mulazzi Davide ^39^, Muraleedharan Visakh ^1^, Murray Lynnette^40^, Mühlan Holger ^111^, Nair Nandesh ^60^, Negru Ancuta ^112^, Nelson David ^1^, Newcombe Virginia ^66^, Nieboer Daan ^47^, Noirhomme Quentin ^95^, Nyirádi József ^5^, Oddo Mauro ^113^, Oldenbeuving Annemarie ^114^, Oresic Matej ^115^, Ortolano Fabrizio ^39^, Palotie Aarno ^116, 117, 118^, Parizel Paul M. ^119^, Patruno Adriana ^120^, Payen Jean-François ^31^, Perera Natascha ^22^, Perlbarg Vincent ^27^, Persona Paolo ^121^, Peul Wilco ^63^, Piippo-Karjalainen Anna ^91^, Pili Floury Sébastien ^122^, Pirinen Matti ^116^, Ples Horia ^112^, Poca Maria Antonia ^123^, Polinder Suzanne ^47^, Pomposo Inigo ^41^, Posti Jussi ^90^, Puybasset Louis ^124^, Radoi Andreea ^123^, Ragauskas Arminas ^125^, Raj Rahul ^91^, Rambadagalla Malinka ^126^, Real Ruben ^53^, Rehorčíková Veronika ^33^, Rhodes Jonathan ^127^, Ripatti Samuli ^116^, Rocka Saulius ^125^, Roe Cecilie ^128^, Roise Olav ^129^, Roks Gerwin ^130^, Rosand Jonathan ^131^, Rosenfeld Jeffrey ^110^, Rosenlund Christina ^132^, Rosenthal Guy ^74^, Rossaint Rolf ^48^, Rossi Sandra ^121^, Rueckert Daniel ^79^, Rusnák Martin ^133^, Sacchi Marco ^17^, Sahakian Barbara ^66^, Sahuquillo Juan ^123^, Sakowitz Oliver ^134, 135^, Sala Francesca ^120^, Sanchez-Porras Renan ^134^, Sandor Janos ^136^, Santos Edgar ^29^, Sasu Luminita ^61^, Savo Davide ^120^, Schäffer Nadine^103^, Schipper Inger ^137^, Schlößer Barbara ^21^, Schmidt Silke ^111^, Schoechl Herbert ^138^, Schoonman Guus ^130^, Schou Rico Frederik ^139^, Schwendenwein Elisabeth ^11^, Schöll Michael ^29^, Sir Özcan ^140^, Skandsen Toril ^141^, Smakman Lidwien ^63^, Smeets Dirk ^101^, Smielewski Peter ^56^, Sorinola Abayomi ^142^, Stamatakis Emmanuel ^66^, Stanworth Simon ^54^, Steinbüchel Nicole ^143^, Stevanovic Ana ^48^, Stevens Robert ^144^, Stewart William ^145^, Steyerberg Ewout W. ^47, 146^, Stocchetti Nino ^147^, Sundström Nina ^36^, Synnot Anneliese ^34, 148^, Taccone Fabio Silvio ^18^, Takala Riikka ^90^, Tamás Viktória ^142^, Tanskanen Päivi ^91^, Taylor Mark Steven ^33^, Te Ao Braden ^71^, Tenovuo Olli ^90^, Telgmann Ralph ^53^, Teodorani Guido ^149^, Theadom Alice ^71^, Thomas Matt ^105^, Tibboel Dick ^42^, Tolias Christos ^150^, Tshibanda Jean-Flory Luaba ^151^, Trapani Tony ^40^, Tudora Cristina Maria ^112^, Vajkoczy Peter ^67^, Vallance Shirley ^43^, Valeinis Egils ^78^, Van der Steen Gregory^60^, van der Jagt Mathieu ^152^, van der Naalt Joukje ^88^, van Dijck Jeroen T.J.M. ^63^, van Essen Thomas A. ^63^, Van Hecke Wim ^101^, van Heugten Caroline ^59^, Van Praag Dominique ^60^, Vande Vyvere Thijs ^101^, Van Waesberghe Julia ^48^, Vanhaudenhuyse Audrey ^27, 95^, Vargiolu Alessia ^120^, Vega Emmanuel ^153^, Velt Kimberley ^47^, Verheyden Jan ^101^, Vespa Paul M. ^154^, Vik Anne ^155^, Vilcinis Rimantas ^156^, Vizzino Giacinta ^17^, Vleggeert-Lankamp Carmen ^63^, Volovici Victor ^82^, Voormolen Daphne ^47^, Vulekovic Peter ^9^, Vámos Zoltán ^69^, Wade Derick ^59^, Wang Kevin K.W. ^157^, Wang Lei ^64^, Wessels Lars ^158^, Wildschut Eno ^42^, Williams Guy ^66^, Wilson Lindsay ^86^, Winkler Maren K.L. ^104^, Wolf Stefan ^158^, Ylén Peter ^159^, Younsi Alexander ^29^, Zaaroor Menashe ^99^, Zhihui Yang ^160^, Ziverte Agate ^78^, Zumbo Fabrizio ^3^.

^1^ Karolinska Institutet, INCF International Neuroinformatics Coordinating Facility, Stockholm, Sweden.

^2^ Division of Neurosurgery, Department of Clinical Neurosciences, Addenbrooke’s Hospital & University of Cambridge, Cambridge, UK.

^3^ Department of Anesthesia & Intensive Care, M. Bufalini Hospital, Cesena, Italy.

^4^ Department of Clinical Neurosciences, Addenbrooke’s Hospital & University of Cambridge, Cambridge, UK.

^5^ János Szentágothai Research Centre, University of Pécs, Pécs, Hungary.

^6^ University of Cincinnati, Cincinnati, Ohio, United States.

^7^ Division of Surgery and Clinical Neuroscience, Department of Physical Medicine and Rehabilitation, Oslo University Hospital and University of Oslo, Oslo, Norway.

^8^ Department of Neurosurgery, University Hospital Northern Norway, Tromso, Norway.

^9^ Department of Neurosurgery, Clinical centre of Vojvodina, Faculty of Medicine, University of Novi Sad, Novi Sad, Serbia.

^10^ Department of Physical Medicine and Rehabilitation, University hospital Northern Norway.

^11^ Trauma Surgery, Medical University Vienna, Vienna, Austria.

^12^ Department of Neurosurgery, Elisabeth-Tweesteden Ziekenhuis, Tilburg, the Netherlands.

^13^ Department of Anesthesiology & Intensive Care, University Hospital Nancy, Nancy, France.

^14^ Riga Eastern Clinical University Hospital, Riga, Latvia.

^15^ Raymond Poincare hospital, Assistance Publique – Hopitaux de Paris, Paris, France.

^16^ Department of Anesthesiology & Intensive Care, S Raffaele University Hospital, Milan, Italy.

^17^ NeuroIntensive Care, Niguarda Hospital.

^18^ Department Anesthesiology and Surgical-Trauma Intensive Care, Hospital Clinic Universitari de Valencia, Spain.

^19^ Department of Neurosurgery, Radboud University Medical Center.

^20^ Department of Neurosurgery, University of Szeged, Szeged, Hungary.

^21^ Institute for Transfusion Medicine (ITM), Witten/Herdecke University, Cologne, Germany.

^22^ International Projects Management, ARTTIC, Munchen, Germany.

^23^ Department of Neurology, Neurological Intensive Care Unit, Medical University of Innsbruck, Innsbruck, Austria.

^24^ Department of Neurosurgery & Anesthesia & intensive care medicine, Karolinska University Hospital, Stockholm, Sweden.

^25^ NIHR Surgical Reconstruction and Microbiology Research Centre, Birmingham, UK.

^26^ Intensive care Unit, CHU Poitiers, Poitiers, France.

^27^ Anesthesie-Réanimation, Assistance Publique – Hopitaux de Paris, Paris, France.

^28^ Department of Anesthesia & ICU, AOU Città della Salute e della Scienza di Torino - Orthopedic and Trauma Center, Torino, Italy.

^29^ Department of Neurosurgery, University Hospital Heidelberg, Heidelberg, Germany.

^30^ Department of Neurology, Odense University Hospital, Odense, Denmark.

^31^ Department of Anesthesiology & Intensive Care, University Hospital of Grenoble, Grenoble, France.

^32^ BehaviourWorks Australia, Monash Sustainability Institute, Monash University, Victoria, Australia.

^33^ Department of Public Health, Faculty of Health Sciences and Social Work, Trnava University, Trnava, Slovakia.

^34^ Quesgen Systems Inc., Burlingame, California, USA.

^35^ Australian & New Zealand Intensive Care Research Centre, Department of Epidemiology and Preventive Medicine, School of Public Health and Preventive Medicine, Monash University, Melbourne, Australia.

^36^ Department of Neurosurgery, Umea University Hospital, Umea, Sweden.

^37^ Department of Neurosurgery, University of Pecs and MTA-PTE Clinical Neuroscience MR Research Group and Janos Szentagothai Research Centre, University of Pecs, Hungarian Brain Research Program, Pecs, Hungary.

^38^ Department of Medical Psychology, Universitätsklinikum Hamburg-Eppendorf, Hamburg, Germany.

^39^ Neuro ICU, Fondazione IRCCS Cà Granda Ospedale Maggiore Policlinico, Milan, Italy.

^40^ ANZIC Research Centre, Monash University, Department of Epidemiology and Preventive Medicine, Melbourne, Vitoria, Australia.

^41^ Department of Neurosurgery, Hospital of Cruces, Bilbao, Spain.

^42^ Intensive Care and Department of Pediatric Surgery, Erasmus Medical Center, Sophia Children’s Hospital, Rotterdam, The Netherlands.

^43^ Department of Neurosurgery, Hospital Universitario 12 de Octubre, Madrid, Spain.

^44^ Department of Neuroscience, Azienda Ospedaliera Università di Padova, Padova, Italy.

^45^ NeuroIntensive Care, ASST di Monza, Monza, Italy.

^46^ School of Medicine and Surgery, Università Milano Bicocca, Milano, Italy.

^47^ Department of Public Health, Erasmus Medical Center-University Medical Center, Rotterdam, The Netherlands.

^48^ Department of Anaesthesiology, University Hospital of Aachen, Aachen, Germany.

^49^ Department of Anesthesia & Neurointensive Care, Cambridge University Hospital NHS Foundation Trust, Cambridge, UK.

^50^ Movement Science Group, Oxford Institute of Nursing, Midwifery and Allied Health Research, Oxford Brookes University, Oxford, UK.

^51^ School of Public Health & PM, Monash University and The Alfred Hospital, Melbourne, Victoria, Australia.

^52^ Radiology/MRI department, MRC Cognition and Brain Sciences Unit, Cambridge, UK.

^53^ Institute of Medical Psychology and Medical Sociology, Universitätsmedizin Göttingen, Göttingen, Germany.

^54^ Oxford University Hospitals NHS Trust, Oxford, UK.

^55^ Department of Neurosurgery, University of Pecs and MTA-PTE Clinical Neuroscience MR Research Group and Janos Szentagothai Research Centre, University of Pecs, Hungarian Brain Research Program (Grant No. KTIA 13 NAP-A-II/8), Pecs, Hungary.

^56^ Brain Physics Lab, Division of Neurosurgery, Dept of Clinical Neurosciences, University of Cambridge, Addenbrooke’s Hospital, Cambridge, UK.

^57^ Intensive Care Unit, CHR Citadelle, Liège, Belgium.

^58^ Intensive Care Unit, CHU, Liège, Belgium.

^59^ Movement Science Group, Faculty of Health and Life Sciences, Oxford Brookes University, Oxford, UK.

^60^ Department of Neurosurgery, Antwerp University Hospital and University of Antwerp, Edegem, Belgium.

^61^ Department of Anesthesia & Intensive Care, Maggiore Della Carità Hospital, Novara, Italy.

^62^ Department of Neurosurgery, University Hospitals Leuven, Leuven, Belgium.

^63^ Dept. of Neurosurgery, Leiden University Medical Center, Leiden, The Netherlands and Dept. of Neurosurgery, Medical Center Haaglanden, The Hague, The Netherlands.

^64^ Department of Neurosurgery, Renji Hospital, Shanghai Jiaotong University School of Medicine, Shanghai, China.

^65^ Department of Neurology, Erasmus MC, Rotterdam, the Netherlands.

^66^ Division of Anaesthesia, University of Cambridge, Addenbrooke’s Hospital, Cambridge, UK.

^67^ Neurologie, Neurochirurgie und Psychiatrie, Charité – Universitätsmedizin Berlin, Berlin, Germany.

^68^ Neurointensive Care, Sheffield Teaching Hospitals NHS Foundation Trust, Sheffield, UK.

^69^ Department of Anaesthesiology and Intensive Therapy, University of Pécs, Pécs, Hungary.

^70^ Departments of Neurology, Clinical Neurophysiology and Neuroanesthesiology, Region Hovedstaden Rigshospitalet, Copenhagen, Denmark.

^71^ National Institute for Stroke and Applied Neurosciences, Faculty of Health and Environmental Studies, Auckland University of Technology, Auckland, New Zealand.

^72^ Department of Medicine, Azienda Ospedaliera Università di Padova, Padova, Italy.

^73^ Department of Anesthesiology and Intensive care, University Hospital Northern Norway, Tromso, Norway.

^74^ Department of Neurosurgery, Hadassah-hebrew University Medical center, Jerusalem, Israel.

^75^ Fundación Instituto Valenciano de Neurorrehabilitación (FIVAN), Valencia, Spain.

^76^ Department of Neurosurgery, Shanghai Renji hospital, Shanghai Jiaotong University/school of medicine, Shanghai, China.

^77^ Emergency Department, CHU, Liège, Belgium.

^78^ Pauls Stradins Clinical University Hospital, Riga, Latvia.

^79^ Department of Computing, Imperial College London, London, UK.

^80^ Lee Kong Chian School of Medicine, Nanyang Technological University, Singapore; and Monash University, Australia.

^81^ Department of Neurosurgery, Neurosciences Centre & JPN Apex trauma centre, All India Institute of Medical Sciences, New Delhi-110,029, India.

^82^ Department of Neurosurgery, Erasmus MC, Rotterdam, the Netherlands.

^83^ Department of Neurosurgery, University of Cincinnati, Cincinnati, Ohio, USA.

^84^ Department of Neurosurgery, Oslo University Hospital, Oslo, Norway.

^85^ Department of Intensive Care Medicine, Radboud University Medical Center.

^86^ Division of Psychology, University of Stirling, Stirling, UK.

^87^ Department of Medical Imaging, St. Olavs Hospital and Department of Neuroscience, Norwegian University of Science and Technology, Trondheim, Norway.

^88^ Department of Neurology, University Medical Center Groningen, Groningen, Netherlands.

^89^ National Institute for Stroke & Applied Neurosciences of the AUT University, Auckland, New Zealand.

^90^ Rehabilitation and Brain Trauma, Turku University Central Hospital and University of Turku, Turku, Finland.

^91^ Helsinki University Central Hospital.

^92^ Hungarian Brain Research Program - Grant No. KTIA 13 NAP-A-II/8, University of Pécs, Pécs, Hungary.

^93^ Department of Intensive Care and Department of Ethics and Philosophy of Medicine, Erasmus Medical Center, Rotterdam, The Netherlands.

^94^ Department of Psichiatry, Clinical centre of Vojvodina, Faculty of Medicine, University of Novi Sad, Novi Sad, Serbia.

^95^ Cyclotron Research Center, University of Liège, Liège, Belgium.

^96^ Emergency Medicine Research in Sheffield, Health Services Research Section, School of Health and Related Research (ScHARR), University of Sheffield, Sheffield, UK.

^97^ Institute of Research in Operative Medicine (IFOM), Witten/Herdecke University, Cologne, Germany.

^98^ VP Global Project Management CNS, ICON, Paris, France.

^99^ Department of Neurosurgery, Rambam Medical Center, Haifa, Israel.

^100^ Department of Anesthesiology & Intensive Care, University Hospitals Southhampton NHS Trust, Southhampton, UK.

^101^ icoMetrix NV, Leuven, Belgium.

^102^ Cambridge University Hospitals, Cambridge, UK.

^103^ Cologne-Merheim Medical Center (CMMC), Department of Traumatology, Orthopedic Surgery and Sportmedicine, Witten/Herdecke University, Cologne, Germany.

^104^ Centrum für Schlaganfallforschung, Charité – Universitätsmedizin Berlin, Berlin, Germany.

^105^ Intensive Care Unit, Southmead Hospital, Bristol, Bristol, UK.

^106^ Department of Neurological Surgery, University of California, San Francisco, California, USA.

^107^ Department of Neurosurgery, CHU, Liège, Belgium.

^108^ Department of Neurosurgery, The Walton centre NHS Foundation Trust, Liverpool, UK.

^109^ Department of Medical Genetics, University of Pécs, Pécs, Hungary.

^110^ National Trauma Research Institute, The Alfred Hospital, Monash University, Melbourne, Victoria, Australia.

^111^ Department Health and Prevention, University Greifswald, Greifswald, Germany.

^112^ Department of Neurosurgery, Emergency County Hospital Timisoara, Timisoara, Romania.

^113^ Centre Hospitalier Universitaire Vaudois.

^114^ Department of Intensive Care, Elisabeth-Tweesteden Ziekenhuis, Tilburg, the Netherlands.

^115^ Department of Systems Medicine, Steno Diabetes Center, Gentofte, Denmark.

^116^ Institute for Molecular Medicine Finland, University of Helsinki, Helsinki, Finland.

^117^ Analytic and Translational Genetics Unit, Department of Medicine; Psychiatric & Neurodevelopmental Genetics Unit, Department of Psychiatry; Department of Neurology, Massachusetts General Hospital, Boston, MA, USA.

^118^ Program in Medical and Population Genetics; The Stanley Center for Psychiatric Research, The Broad Institute of MIT and Harvard, Cambridge, MA, USA.

^119^ Department of Radiology, Antwerp University Hospital and University of Antwerp, Edegem, Belgium.

^120^ NeuroIntenisve Care Unit, Department of Anesthesia & Intensive Care, ASST di Monza, Monza, Italy.

^121^ Department of Anesthesia & Intensive Care, Azienda Ospedaliera Università di Padova, Padova, Italy.

^122^ Intensive Care Unit, CHRU de Besançon, Besançon, France.

^123^ Department of Neurosurgery, Vall d’Hebron University Hospital, Barcelona, Spain.

^124^ Department of Anesthesiology and Critical Care, Pitié -Salpêtrière Teaching Hospital, Assistance Publique, Hôpitaux de Paris and University Pierre et Marie Curie, Paris, France.

^125^ Department of Neurosurgery, Kaunas University of technology and Vilnius University, Vilnius, Lithuania.

^126^ Rezekne Hospital, Latvia.

^127^ Department of Anesthesia, Critical Care & Pain Medicine NHS Lothian & University of Edinburg, Edinburgh, UK.

^128^ Department of Physical Medicine and Rehabilitation, Oslo University Hospital/University of Oslo, Oslo, Norway.

^129^ Division of Surgery and Clinical Neuroscience, Oslo University Hospital, Oslo, Norway.

^130^ Department of Neurology, Elisabeth-TweeSteden Ziekenhuis, Tilburg, the Netherlands.

^131^ Broad Institute, Cambridge MA Harvard Medical School, Boston MA, Massachusetts General Hospital, Boston MA, USA.

^132^ Department of Neurosurgery, Odense University Hospital, Odense, Denmark.

^133^ International Neurotrauma Research Organization, Vienna, Austria.

^134^ Klinik für Neurochirurgie, Klinikum Ludwigsburg, Ludwigsburg, Germany.

^135^ University Hospital Heidelberg, Heidelberg, Germany.

^136^ Division of Biostatistics and Epidemiology, Department of Preventive Medicine, University of Debrecen, Debrecen, Hungary.

^137^ Department of Traumasurgery, Leiden University Medical Center, Leiden, The Netherlands.

^138^ Department of Anaesthesiology and Intensive Care, AUVA Trauma Hospital, Salzburg, Austria.

^139^ Department of Neuroanesthesia and Neurointensive Care, Odense University Hospital, Odense, Denmark.

^140^ Department of Emergency Care Medicine, Radboud University Medical Center.

^141^ Department of Physical Medicine and Rehabilitation, St. Olavs Hospital and Department of Neuroscience, Norwegian University of Science and Technology, Trondheim, Norway.

^142^ Department of Neurosurgery, University of Pécs, Pécs, Hungary.

^143^ Universitätsmedizin Göttingen, Göttingen, Germany.

^144^ Division of Neuroscience Critical Care, John Hopkins University School of Medicine, Baltimore, USA.

^145^ Department of Neuropathology, Queen Elizabeth University Hospital and University of Glasgow, Glasgow, UK.

^146^ Dept. of Department of Biomedical Data Sciences, Leiden University Medical Center, Leiden.

^147^ Department of Pathophysiology and Transplantation, Milan University, and Neuroscience ICU, Fondazione IRCCS Cà Granda Ospedale Maggiore Policlinico, Milano, Italy.

^148^ Cochrane Consumers and Communication Review Group, Centre for Health Communication and Participation, School of Psychology and Public Health, La Trobe University, Melbourne, Australia.

^149^ Department of Reahabilitation, M. Bufalini Hospital, Cesena, Italy.

^150^ Department of Neurosurgery, Kings college London, London, UK.

^151^ Radiology/MRI Department, CHU, Liège, Belgium.

^152^ Department of Intensive Care, Erasmus MC, Rotterdam, the Netherlands.

^153^ Department of Anesthesiology-Intensive Care, Lille University Hospital, Lille, France.

^154^ Director of Neurocritical Care, University of California, Los Angeles, USA.

^155^ Department of Neurosurgery, St. Olavs Hospital and Department of Neuroscience, Norwegian University of Science and Technology, Trondheim, Norway.

^156^ Department of Neurosurgery, Kaunas University of Health Sciences, Kaunas, Lithuania.

^157^ Department of Psychiatry, University of Florida, Gainesville, Florida, USA.

^158^ Interdisciplinary Neuro Intensive Care Unit, Charité – Universitätsmedizin Berlin, Berlin, Germany.

^159^ VTT Technical Research Centre, Tampere, Finland.

^160^ University of Florida, Gainesville, Florida, USA

## References

[1] Albuquerque FC (2013). Intracranial pressure monitoring after blunt head injuries: conflicting opinions. World Neurosurg.

[2] Bosco JLF, Silliman RA, Thwin SS, Geiger AM, Buist DSM, Prout MN, Yood MU, Haque R, Wei F, Lash TL (2010). A most stubborn bias: no adjustment method fully resolves confounding by indication in observational studies. J Clin Epidemiol.

[3] Bragge P, Synnot A, Maas AI, Menon DK, Cooper DJ, Rosenfeld JV, Gruen RL (2016). A state-of-the-science overview of randomized controlled trials evaluating acute management of moderate-to-severe traumatic brain injury. J Neurotrauma.

[4] Brattheim BJ, Eikemo TA, Altreuther M, Landmark AD, Faxvaag A (2012). Regional disparities in incidence, handling and outcomes of patients with symptomatic and ruptured abdominal aortic aneurysms in Norway. Eur J Vasc Endovasc Surg.

[5] Bullock MR, Chesnut R, Ghajar J, Gordon D, Hartl R, Newell DW, Servadei F, Walters BC, Wilberger JE (2006). Introduction. Neurosurgery.

[6] Carney N, Totten AM, OʼReilly C, et al (2016) Guidelines for the management of severe traumatic brain injury, Fourth Edition. Neurosurgery 1 10.1227/NEU.0000000000001432 27654000

[7] Chesnut RM, Temkin N, Carney N (2012). A trial of intracranial-pressure monitoring in traumatic brain injury. N Engl J Med.

[8] Cnossen MC, Huijben JA, van der Jagt M, et al (2017) Variation in monitoring and treatment policies for intracranial hypertension in traumatic brain injury: a survey in 66 neurotrauma centers participating in the CENTER-TBI study. Crit Care 21(1):233 10.1186/s13054-017-1816-9 PMC5586023 28874206

[9] Cnossen MC, Polinder S, Lingsma HF, Maas AIR, Menon D, Steyerberg EW, CENTER-TBI Investigators and Participants (2016) Variation in structure and process of care in traumatic brain injury: provider profiles of European Neurotrauma centers participating in the CENTER-TBI study. PLoS One 11(8):e0161367 10.1371/journal.pone.0161367 PMC5003388 27571205

[10] Cnossen MC, van Essen TA, Ceyisakar IE (2018). Adjusting for confounding by indication in observational studies: a case study in traumatic brain injury. Clinical Epidemiology.

[11] Compagnone C, Murray GD, Teasdale GM, Maas AIR, Esposito D, Princi P, D Avella D, Servadei F (2005) The management of patients with Intradural post-traumatic mass lesions: a multicenter survey of current approaches to surgical management in 729 patients coordinated by the European Brain Injury Consortium. Neurosurgery:1183–1192 10.1227/01.neu.0000279218.53504.fe 18813166

[12] Cooper DJ, Nichol A, Hodgson C (2016). Craniectomy for traumatic intracranial hypertension. N Engl J Med.

[13] Ergina PL, Cook JA, Blazeby JM, Boutron I, Clavien P-A, Reeves BC, Seiler CM (2009). Challenges in evaluating surgical innovation. Lancet.

[14] Foks KA, Cnossen MC, Dippel DWJ, Maas A, Menon D, van der Naalt J, Steyerberg EW, Lingsma H, Polinder S (2017) Management of mild traumatic brain injury at the emergency department and hospital admission in Europe: a survey of 71 neurotrauma centers participating in the CENTER-TBI study. J Neurotrauma. 10.1089/neu.2016.4919 10.1089/neu.2016.4919 28398105

[15] Ghajar J, Carney N (2013). Intracranial-pressure monitoring in traumatic brain injury. N Engl J Med.

[16] Gregson BA, Mendelow AD (2003). International variations in surgical practice for spontaneous intracerebral hemorrhage. Stroke.

[17] Hesdorffer DC, Ghajar J (2007). Marked improvement in adherence to traumatic brain injury guidelines in United States trauma centers. The Journal of Trauma: Injury, Infection, and Critical Care.

[18] Huijben JA, van der Jagt M, Cnossen MC (2017). Variation in blood transfusion and coagulation management in traumatic brain injury at the intensive care unit: a survey in 66 neurotrauma centers participating in the collaborative European NeuroTrauma Effectiveness Research in Traumatic Brain Injury (CENTER-TBI) study. Journal of Neurotrauma neu.

[19] Hutchinson PJ, Kolias AG, Timofeev IS (2016). Trial of decompressive craniectomy for traumatic intracranial hypertension. N Engl J Med.

[20] Kahle KT, Duhaime A-C (2013). Intracranial-pressure monitoring in traumatic brain injury. N Engl J Med.

[21] Kolias AG, Scotton WJ, Belli A (2013). Surgical management of acute subdural haematomas: current practice patterns in the United Kingdom and the Republic of Ireland. Br J Neurosurg.

[22] Li LM, Kolias AG, Guilfoyle MR, Timofeev I, Corteen EA, Pickard JD, Menon DK, Kirkpatrick PJ, Hutchinson PJ (2012). Outcome following evacuation of acute subdural haematomas: a comparison of craniotomy with decompressive craniectomy. Acta Neurochir.

[23] Lingsma HF, Roozenbeek B, Li B, Lu J, Weir J, Butcher I, Marmarou A, Murray GD, Maas AIR, Steyerberg EW (2011). Large between-center differences in outcome after moderate and severe traumatic brain injury in the international mission on prognosis and clinical trial design in Traumatic Brain Injury (IMPACT) study. Neurosurgery.

[24] Maas AIR, Menon DK, Lingsma HF, Pineda JA, Sandel ME, Manley GT (2012). Re-orientation of clinical research in traumatic brain injury: report of an international workshop on comparative effectiveness research. J Neurotrauma.

[25] Maas AIR, Menon DK, Steyerberg EW, Citerio G, Lecky F, Manley GT, Hill S, Legrand V, Sorgner A (2015). Collaborative European NeuroTrauma effectiveness research in traumatic brain injury (CENTER-TBI): a prospective longitudinal observational study. Neurosurgery.

[26] Maas AIR, Stocchetti N, Bullock R (2008). Moderate and severe traumatic brain injury in adults. The Lancet Neurology.

[27] Macefield RC, Boulind CE, Blazeby JM (2014). Selecting and measuring optimal outcomes for randomised controlled trials in surgery. Langenbeck's Arch Surg.

[28] Mattei TA (2013). Intracranial pressure monitoring in severe traumatic brain injury: who is still bold enough to keep sinning against the level I evidence?. World Neurosurg.

[29] Mendelow AD, Gregson BA, Rowan EN, Francis R, McColl E, McNamee P, Chambers I, Unterberg AW, Boyers D, Mitchell P (2015) Early surgery versus initial conservative treatment in patients with traumatic intracerebral haemorrhage [STITCH (trauma)]: the first randomised trial. J Neurotrauma. 10.1089/neu.2014.3644 10.1089/neu.2014.3644 PMC4545564 25738794

[30] Murray GD, Teasdale GM, Braakman R (1999). The European brain injury consortium survey of head injuries. Acta Neurochir.

[31] Rayan N, Barnes S, Fleming N, Kudyakov R, Ballard D, Gentilello LM, Shafi S (2012) Barriers to compliance with evidence-based care in trauma. J Trauma Acute Care Surg 72(3):585–92– discussion 592–3 10.1097/TA.0b013e318243da4d 22491540

[32] Servadei F, Compagnone C, Sahuquillo J (2007). The role of surgery in traumatic brain injury. Curr Opin Crit Care.

[33] Timmons SD, Toms SA (2012). Comparative effectiveness research in neurotrauma. Neurosurg Focus.

[34] van Essen TA, de Ruiter GCW, Kho KH, Peul WC (2016) Neurosurgical treatment variation of traumatic brain injury: evaluation of acute subdural hematoma management in Belgium and the Netherlands. J Neurotrauma doi: 10.1089/neu.2016.4495 10.1089/neu.2016.4495 27393190

[35] Van Essen TA, Dijkman M, Cnossen MC, Moudrous W, Ardon H, Schoonman GG, Steyerberg EW, Peul W, Lingsma H, de Ruiter GCW (2018) Comparative effectiveness of surgery for traumatic acute subdural hematoma in an aging population. J Neurotrauma. 10.1089/neu.2018.5869 10.1089/neu.2018.5869 30234429

