# Supplementary material for: Variation in neurosurgical management of traumatic brain injury: a survey in 68 centers participating in the CENTER-TBI study
Source: Acta Neurochir (Wien). 2018 Dec 19;161(3):435–49. doi: 10.1007/s00701-018-3761-z (PMC6407836; doi:10.1007/s00701-018-3761-z)
Supplement: Supplementary file 1 — Questionnaire neurosurgery. (PDF 158 kb) [file 701_2018_3761_MOESM1_ESM.pdf]

Questionnaire 7: Neurosurgery

Patient Identification Information

For a printable form to fill out
Click Here

Please, save your answers frequently by pressing the save buttons placed throughout the questionnaire.

This questionnaire can be completed by a neurosurgeon, trauma surgeon or other surgeon who performs surgery in TBI patients

For the completion of this questionnaire, we advise you to ask help from a data manager, administrative staff member and/or someone from the financial department in your hospital, since we ask for hospital data in this questionnaire. It is very important that this information is accurate, and searched for in annual reports, registries and other data sources rather than estimated.

This questionnaire also includes questions about the general policy in your hospital. The responses to these questions should represent, as best as practicable, a general consensus on treatment at your centre, rather than individual management preferences. Consequently, you should provide responses that describe not what you would do personally, but how the majority of patients would generally be treated in your centre.

There are no ‘right’ or ‘wrong’ answers so please give us a realistic and honest view of how the care in your hospital is organized. Your answers will only be used to answer the scientific questions in CENTER-TBI and no information in any form will be reported on individual centre level. Some of the questions may seem similar, but please answer all questions.

If you have any questions or problem, please contact:
Maryse Cnossen, PhD student m.c.cnossen@erasmusmc.nl

Start Date Time

End Date Time

Information about the completer of the questionnaire

Other than the CENTER-TBI investigator, which of the following individuals was involved in completion of this questionnaire? *Select all that apply*

- ☐ Neurologist
- ☐ Neurosurgeon
- ☐ Trauma Surgeon
- ☐ Emergency Department (ED) physician
- ☐ Administrative staff member / data manager / financial department
- ☐ Other Please specify other:

☐ NA. The questionnaire was completed solely by the CENTER-TBI local investigator

The Local investigator is the senior clinician(s) at your hospital involved in supervision of CENTER-TBI

Volume
1. In the year 2013, how many Traumatic Brain Injury (TBI) related surgeries have been performed with the following diagnoses?
Please find the exact number of surgeries performed in your annual report / registry

	Number
Acute subdural hematoma	
Contusion/traumatic intracerebral hematoma	
Decompressive craniectomy for TBI – hemicraniectomy	
Decompressive craniectomy for TBI – bifrontal	
Decompressive craniectomy for TBI – removal previous bone flap	
Depressed skull fracture	
Epidural hematoma	
Ventriculostomy In TBI patients	
Cranioplasty	

Where did you find this information?

Name the source: for example annual report, registry

Staffing

2. How many neurosurgeons (in FTE) work at your hospital?

☐ FTE neurosurgeons

☐ FTE neurosurgery trainees in residency training

☐ FTE neurosurgery trainees not in residency training

FTE = Full time equivalent. '1 FTE' may be constituted by one person who works on a fulltime basis, but can also refer to two persons who work half-time.

The amount of FTEs do not have to be a whole number. If the amount of FTE is, for example, 3.3, please write down '3.3' here and not '3'!

If there are persons with out of hours work that is contracted and paid for, you can count them as > 1 FTE. For example, if there is a physician that is paid for 60 hours a week and 48 hours a week is considered as a FTE for a doctor in your hospital, you can count this physician as 60/48 = 1.25 FTE

The term 'trainee not in residency training' refers to a clinician working in your hospital who is not qualified as a specialist, but is also not part of a formal training scheme towards becoming a specialist (neurosurgeon in this case).

3. How many trauma surgeons (in FTE) work at your hospital?

☐ FTE trauma surgeons

☐ FTE trauma surgeon trainees in residency training

☐ FTE trauma surgeon trainees not in residency training

FTE = Full time equivalent. '1 FTE' may be constituted by one person who works on a fulltime basis, but can also refer to two persons who work half-time.

The amount of FTEs do not have to be a whole number. If the amount of FTE is, for example, 3.3, please write down '3.3'

here and not ‘3’!

If there are persons with out of hours work that is contracted and paid for, you can count them as > 1 FTE. For example, if there is a physician that is paid for 60 hours a week and 48 hours a week is considered as a FTE for a doctor in your hospital, you can count this physician as 60/48 = 1.25 FTE

The term ‘trainee not in residency training’ refers to a clinician working in your hospital who is not qualified as a specialist, but is also not part of a formal training scheme towards becoming a specialist (trauma surgeon in this case).

In this response, the term ‘trauma surgeon’ refers to an individual who specializes in trauma surgery, not a general surgeon or orthopaedic surgeon who happens to perform damage control surgery as part of wider responsibilities

4. Treatment decisions regarding neurosurgical interventions in Traumatic Brain Injury (TBI) patients in your Intensive Care Unit (ICU) are mainly determined by:

- ☐ Trauma surgeon
- ☐ Neurosurgeon
- ☐ General surgeon or orthopaedic surgeon
- ☐ Neurointensivist
- ☐ Neurologist
- ☐ General intensivists

Only tick the person(s) that is responsible for the decision. It is possible that options are discussed in a multidisciplinary team, but for this question we want to know who is finally responsible for the decision. In this response, the term ‘trauma surgeon’ refers to an individual who specializes in trauma surgery, not a general surgeon or orthopaedic surgeon who happens to perform damage control surgery as part of wider responsibilities

5. In your hospital, what surgical discipline can be involved in the most urgent cranial surgical interventions in patients with life threatening intracranial space-occupying lesions (sometimes referred to as “super emergency” situations)?
Intracranial surgery does not include ICP monitor placement

Please use the following scale:
Never (0-10%)
Rarely (10-30%)
Sometimes (30-70%)
Frequently (70-90%)
Always (90-100%)

Neurosurgeon	○ Never	○ Rarely	○ Sometimes	○ Frequently	○ Always
Trauma	○ Never	○ Rarely	○ Sometimes	○ Frequently	○ Always
General / orthopaedic surgeon	○ Never	○ Rarely	○ Sometimes	○ Frequently	○ Always
Other surgeon	○ Never	○ Rarely	○ Sometimes	○ Frequently	○ Always

Other, please specify:

A super-emergency situation refers to a situation in which a patients needs immediate TBI related surgery (knife to skin within 1 hour of arrival in the hospital). In this response, the term ‘trauma surgeon” refers to an individual who specializes in trauma surgery, not a general surgeon or orthopaedic surgeon who happens to perform damage control surgery as part of wider responsibilities

6. In your hospital, is there 24/7 qualified neurosurgical coverage?
Select all that apply

- ☐ No
- ☐ There is 24/7 in-house availability of a qualified neurosurgeon
- ☐ There is 24/7 in-house availability of a neurosurgical trainee in residency training
- ☐ Qualified neurosurgeons are on call and will arrive within 30 minutes
- ☐ Neurosurgical trainees in residency training are on call and will arrive within 30 minutes

☐ Qualified neurosurgeons are on call and will arrive in more than 30 minutes
☐ There is 24/7 access to a qualified neurosurgeon by telecommunication / phone. Qualified neurosurgeons are however not 24/7 in-house or on call
☐ Other
Please specify other:

7. What is the general policy with regard to management of extremity (limb) fractures in patients with severe Traumatic Brain Injury (TBI) ?

◯ Damage control: We focus on the TBI. All extremity fractures are stabilized, but definitive treatment delayed
◯ Definitive care: We try to operate/fixate the extremity fractures as soon as possible

The responses to this question should represent, as best as practicable, a general consensus on treatment at your centre, rather than individual management preferences.

8. Do you have guidelines, protocols or policy documents about this topic?

◯ No
◯ Yes, in favour of damage control
◯ Yes, in favour of definitive care

Surgical management of traumatic intracranial mass lesions

Again, the following questions should represent a general consensus on treatment at your centre, rather than individual management preferences.

9. How is the size of a focal post-traumatic mass lesion estimated on CT?
Select all that apply
☐ Based on visual intuition by the neurosurgeon (e.g. no actual measurement)
☐ Based on width, diameter and/or amount of midline shift of the mass lesion
☐ Based on volume measurements with imaging software
☐ Based on volume measurements with direct calculation (e.g. the formula for an ellipsoid)
☐ Other
Please specify other:

10. Are the Brain Trauma Foundation guidelines followed for:	Please use the following scale:<br>Never (0-10%)<br>Rarely (10-30%)<br>Sometimes (30-70%)<br>Frequently (70-90%)<br>Always (90-100%)
Epidural hematoma (EDH) management?	◯ Never ◯ Rarely ◯ Sometimes ◯ Frequently ◯ Always
Acute Subdural hematoma (SDH) management?	◯ Never ◯ Rarely ◯ Sometimes ◯ Frequently ◯ Always
Management of intraparenchymal mass lesions (contusions)?	◯ Never ◯ Rarely ◯ Sometimes ◯ Frequently ◯ Always

Surgical management of acute SDH

11. Is the decision on surgery in acute SDH influenced by age?	◯ Never ◯ Rarely ◯ Sometimes ◯ Frequently ◯ Always

12. Are there acute SDH volume/ thickness thresholds above which your protocol/institutional practice advises surgery (i.e. evacuation)?

◯ No
◯ Yes

If yes, which minimum volume or thickness is used as threshold to operate?

13. Can decompressive craniectomy be added to the surgical evacuation of the acute SDH?

- Yes, standard and routinely in every patient
- Yes, but dependent on intraoperative findings
- No, sometimes delayed in a second procedure in case of uncontrollable ICP
- No, never

Surgical management of intraparenchymal mass lesions (contusions)

14. The general policy in your institute for management of intraparenchymal mass lesions (contusions) is:

- Pre-emptive surgery to prevent deterioration
- Delayed surgery only after deterioration including intracranial hypertension
- Variable, depending on surgeon
- Other

Please specify other:

15. Can decompressive craniectomy be added to the surgical evacuation of the intraparenchymal hematoma?

- Yes, standard and routinely in every patient
- Yes, but dependent on intraoperative findings
- No, sometimes delayed in a second procedure in case of uncontrollable ICP
- No, never

Please use the following scale:
Never (0-10%)
Rarely (10-30%)
Sometimes (30-70%)
Frequently (70-90%)
Always (90-100%)

16. In case of refractory intracranial hypertension / pressure, how often do you use decompressive craniectomy in patients with severe Traumatic Brain Injury (TBI)?

Never Rarely Sometimes Frequently Always

16b. When is decompressive craniectomy mostly employed?

- Early – within 6-12 hours of refractory ICP
- Later – as a last effort to control ICP

17. Are there ICP thresholds (eg > 20, 25, 30 mm Hg), above which you would consider delayed decompressive craniectomy?

- No, I would never perform a decompressive craniectomy
- No, I do not use ICP values in the decision to perform a decompressive craniectomy
- Yes

The responses to this question should represent, as best as practicable, a general consensus on treatment at your centre, rather than individual management preferences.

If yes, which threshold

If yes, which other patient related parameters do you use to decide upon performing a decompressive craniectomy?

Please list or state 'None'

If no I do not use ICP values in the decision to perform a decompressive craniectomy: What patient related parameters do you use to decide upon performing a decompressive craniectomy?

18. What are indications for decompressive craniotomy?
Select all that apply
- [ ] Pre-emptive approach to treatment of (suspected) raised ICP (not last resort)
- [ ] Raised ICP, refractory to medical management (last resort)
- [ ] ICP not monitored, but CT evidence of raised ICP
- [ ] Not directly planned, but decided on because of intra-operative brain swelling
- [ ] Routinely performed with every acute SDH or contusion evacuation

Neurosurgical decision making
19. Is there structural variation between (neuro)surgeons within your hospital with regard to the decision to place an ICP sensor?
○ No
○ Yes

Structural variation refers to a situation in which one or more of the neurosurgeons are generally more likely to place an ICP sensor than others.

20. Is there structural variation between (neuro)surgeons within your hospital with regard to the decision to evacuate a mass lesion?
○ No
○ Yes
○ Both, depending on what type of mass lesion
Please elaborate:

21. What factors influence surgical decision-making in patients with Traumatic Brain Injury (TBI)?

Please use the following scale:
Never (0-10%)
Rarely (10-30%)
Sometimes (30-70%)
Frequently (70-90%)
Always (90-100%)

Age of the patient	○ Never	○ Rarely	○ Sometimes	○ Frequently	○ Always
Pupillary size and responses	○ Never	○ Rarely	○ Sometimes	○ Frequently	○ Always
Time of the day	○ Never	○ Rarely	○ Sometimes	○ Frequently	○ Always
Time from trauma to ED	○ Never	○ Rarely	○ Sometimes	○ Frequently	○ Always
Persistent high ICP	○ Never	○ Rarely	○ Sometimes	○ Frequently	○ Always
Guidelines / protocols	○ Never	○ Rarely	○ Sometimes	○ Frequently	○ Always
Expertise of the surgeon	○ Never	○ Rarely	○ Sometimes	○ Frequently	○ Always
Opinion of other disciplines (.e.g intensivists, nurses)	○ Never	○ Rarely	○ Sometimes	○ Frequently	○ Always
Whether the TBI was a consequence of a suicide attempt	○ Never	○ Rarely	○ Sometimes	○ Frequently	○ Always
Other	○ Never	○ Rarely	○ Sometimes	○ Frequently	○ Always

Other, please specify:

*** End of List ***
