# Supplementary figures and images for: Variation in neurosurgical management of traumatic brain injury: a survey in 68 centers participating in the CENTER-TBI study
Source: Acta Neurochir (Wien). 2018 Dec 19;161(3):435–49. doi: 10.1007/s00701-018-3761-z (PMC6407836; doi:10.1007/s00701-018-3761-z)

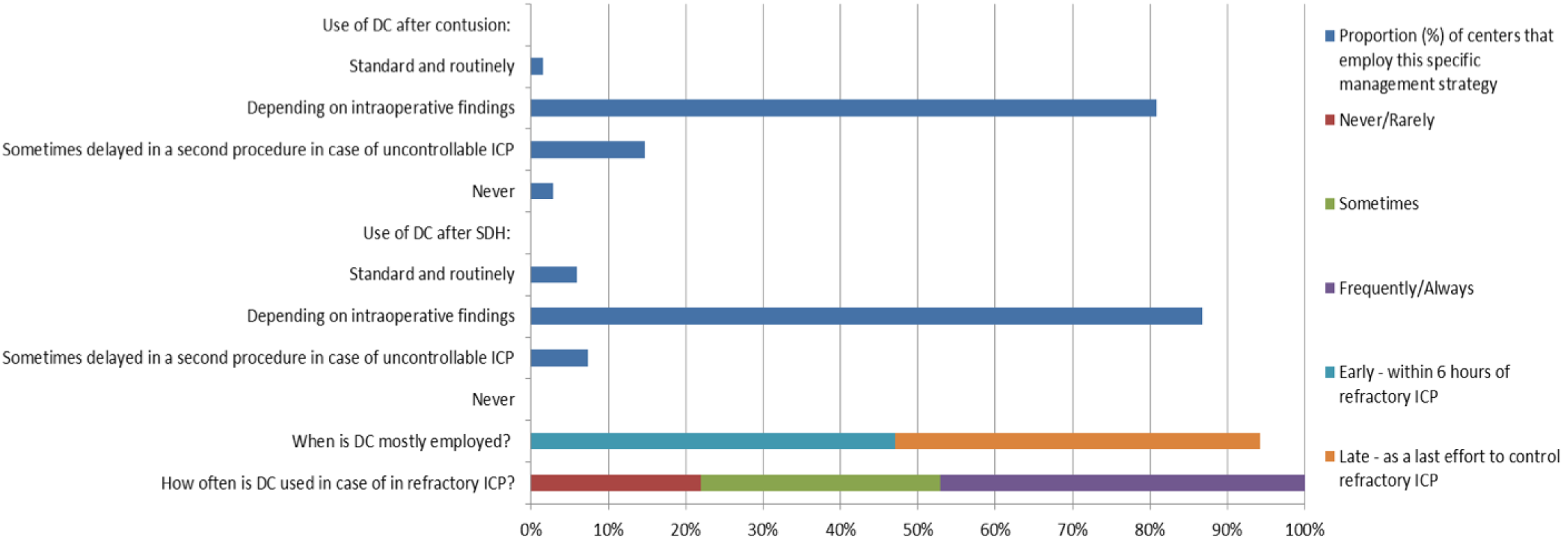

Supplement: Supplementary file 2 — The use of a decompressive craniectomy. (PNG 651 kb) [file 701_2018_3761_Fig5_ESM.png]

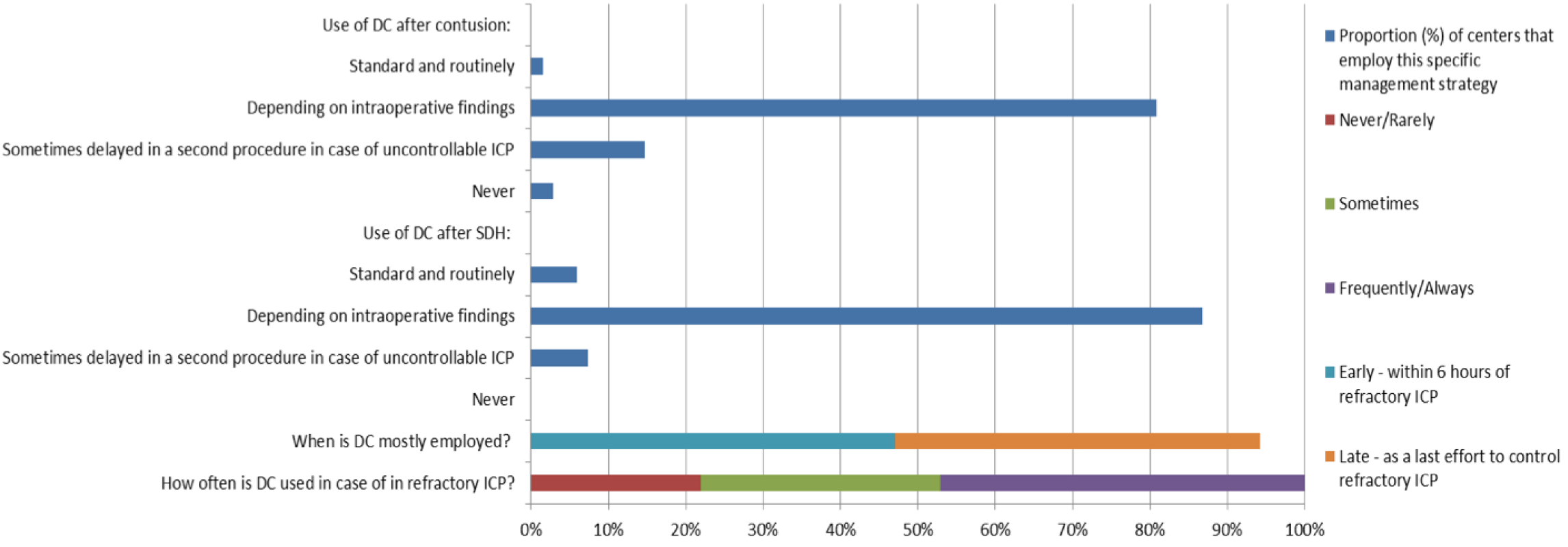

Supplement: Supplementary file 3 — High resolution image (TIF 5387 kb) [file 701_2018_3761_MOESM2_ESM.tif]
